# The development and pilot testing of a behavioral activation-based treatment for depressed mood and multiple health behavior change in patients with recent acute coronary syndrome

**DOI:** 10.1371/journal.pone.0261490

**Published:** 2022-02-03

**Authors:** Emily C. Gathright, Katherine Diaz Vickery, Woubeshet Ayenew, Matthew C. Whited, Melissa Adkins-Hempel, Michelle Chrastek, Jill K. Carter, Rochelle K. Rosen, Wen-Chih Wu, Andrew M. Busch

**Affiliations:** 1 Center for Behavioral and Preventive Medicine, The Miriam Hospital, Providence, Rhode Island, United States of America; 2 Department of Psychiatry and Human Behavior, Alpert Medical School of Brown University, Providence, Rhode Island, United States of America; 3 Health, Homelessness and Criminal Justice Lab, Hennepin Healthcare Research Institute, Minneapolis, Minnesota, United States of America; 4 Department of Medicine, Hennepin Healthcare, Minneapolis, Minnesota, United States of America; 5 Department of Medicine, University of Minnesota, Minneapolis, Minnesota, United States of America; 6 Department of Psychology, East Carolina University, Greenville, North Carolina, United States of America; 7 Behavioral Health Equity Research Group, Hennepin Healthcare Research Institute, Minneapolis, Minnesota, United States of America; 8 Department of Behavioral and Social Sciences, Brown School of Public Health, Brown University, Providence, Rhode Island, United States of America; 9 Division of Cardiology, Providence Veterans Affairs Medical Center, Providence, Rhode Island, United States of America; 10 Lifespan Cardiovascular Institute, The Miriam Hospital, Providence, Rhode Island, United States of America; 11 Department of Medicine, Alpert Medical School of Brown University, Providence, Rhode Island, United States of America; Prince Sattam Bin Abdulaziz University, College of Applied Medical Sciences, SAUDI ARABIA

## Abstract

**Background:**

Patients with acute coronary syndrome (ACS) with depressed mood demonstrate poor cardiovascular behavioral risk profiles and elevated risk for recurrent ACS and mortality. Behavioral Activation (BA) offers an intervention framework for an integrated treatment targeting both depression and critical health behaviors post-ACS. Behavioral Activation for Health and Depression (BA-HD) was developed and pilot tested in a multiphase iterative process.

**Methods:**

First, an initial treatment manual was conceptualized based on the team’s prior work, as well as the extant literature. Second, qualitative interviews were conducted with target patients and target providers on the proposed BA-HD treatment rationale, content, and structure. Framework matrix analyses were used to summarize and aggregate responses. Third, an expert panel was convened to elicit additional manual refinements. Finally, patients with post-ACS depression and health behavior non-adherence were recruited to complete an open pilot trial to evaluate acceptability (Client Satisfaction Questionnaire [CSQ], exit interview) and treatment engagement (number of sessions attended; treatment completion was considered completion of 8 out of 10 possible sessions).

**Results:**

The initial BA-HD treatment manual expanded an existing treatment manual for post-ACS BA-based mood management and smoking cessation to target four health behaviors relevant to post-ACS patients (e.g., smoking cessation, medication adherence, physical activity, and diet). After the initial conceptualization, ten post-ACS patients and eight cardiac rehabilitation professionals completed qualitative interviews. Patients endorsed bi-directional interactions between mood and health behaviors post-ACS. Both patients and providers expressed general support of the proposed treatment rationale and values-guided, collaborative goal-setting approach. Patients, providers, and experts provided feedback that shaped the iterative manual development. After the BA-HD manual was finalized, eight participants were enrolled in a single-arm pilot trial. The mean CSQ score was 30.57 ± 2.23, indicating high satisfaction. Seven out of eight (88%) completed treatment. Pre- to post treatment improvements in depressed mood and health behaviors were promising.

**Conclusions:**

BA-HD treatment is an acceptable approach to target both mood and health behaviors in post-ACS patients with depression. A future larger, controlled trial is needed to evaluate the efficacy of the BA-HD treatment.

**Trial registration:**

ClinicalTrials.gov Identifier: NCT04158219

## Introduction

Over one million hospital discharges following acute coronary syndrome (ACS; ST-elevation myocardial infarction [STEMI], non-ST elevation myocardial infarction [N-STEMI], unstable angina) occur each year in the US [1]. Modifiable behavioral risk factors precede both initial ACS as well as increase risk for subsequent ACS events [2, 3]. In the wake of an ACS event, patients are instructed to follow lifestyle recommendations that span multiple domains including abstinence from tobacco, medication adherence, physical activity, and healthful dietary habits.

Depression is common following ACS with approximately 20% of patients meeting criteria for clinical depression [4]. Depressive symptoms interfere with health behavior engagement [5] and predict subsequent morbidity and mortality [6]. Specifically, adults post-ACS with depression have higher rates of smoking and lower motivation to quit smoking [7], higher medication non-adherence, lower physical activity, and less healthful diet relative to adults post-ACS without depression [8].

Use of health behavior change techniques post-ACS without taking into account the context of depression symptoms may limit optimal progress. For example, depression symptoms such as anhedonia and low motivation can limit efforts to initiate activity, including health-promoting behaviors. For some patients, a compounding cycle may evolve such that lack of health behavior engagement engenders or exacerbates feelings of guilt and low self-worth. Conversely, treatment of post-ACS depression alone is not sufficient to improve behavioral risk factors [9]. Because of the indirect and direct associations between depression, health behavior engagement, and cardiac outcomes post-ACS, a treatment that targets both depressed mood *and* relevant behavioral risk factors may be an effective and efficient approach for reducing risk. However, to date, most treatments have targeted mood and cardiac health behaviors independently. Given the important relationships between depression and cardiac health behaviors, consideration of an integrated treatment approach is warranted. Such an approach has been specifically recommended by experts (e.g., Roy 2019 [10]).

Behavioral Activation (BA) is well-suited to serve as a framework for an integrated mood and risk factor treatment. BA is a counseling intervention for mood management with strong evidence for moderate to large improvements in depressed mood [11]. BA takes a collaborative goal-setting approach whereby patients and providers identify personalized “activation goals” that will increase a patient’s contact with long term sources of positive reinforcement. All activation goals are designed to be reasonable for the patient to achieve in order to maximize success. To generate activation goals that maximize contact with sources of positive reinforcement, BA can include an exploration of patients’ individual values, which are the patients’ personal definitions of how patients wish to live their lives in a certain area [12]. BA has shown efficacy for depression in medical patients (e.g., Choi et al. 2020 [13]) and preliminary efficacy as a combination treatment for depression and health behavior change (e.g., Tull 2018 [14]; Ali, 2020 [15]; Martinez-Vispo, 2019 [16]). Further, our group has demonstrated preliminary support for the feasibility, acceptability, and efficacy of an integrated behavioral activation for mood management and smoking cessation counseling following ACS hospitalization [17].

The objective of the present research was to expand the treatment targets of our prior work to include multiple health behaviors known to be critical for improving cardiac health post-ACS through formative qualitative work and pilot testing. We chose smoking, medication adherence, physical activity, and diet as target health behaviors because there is specific evidence suggesting that these behaviors are associated with morbidity and mortality, are worsened in the context of depression, and at least partially mediate the relationship between depression and morbidity and mortality [18–20].

The team sought to develop a feasible and acceptable treatment manual (BA for Health and Depression [BA-HD]) via four phases: (1) conceptualization of the initial manual framework, (2) formative intervention development via qualitative interviews with target patients and target providers, (3) elicitation of additional feedback from an expert panel, and (4) pilot testing including manual demonstration by a clinical psychologist (AMB) and a small open trial of the initial BA-HD treatment manual conducted by a target provider (JKC). See Table 1.

**Table 1:** Treatment development process.

Phase of Treatment Development	Description of Study Phase	
Phase 1: Conceptualization	Initial manual development	Iterative manual refinement
Phase 2: Formative intervention development	Qualitative interviews with target patients and providers	
Phase 3: Expert panel	Additional feedback elicitation from content experts	
Phase 4: Pilot test	Manual demonstration and open trial with post-treatment qualitative feedback	

## Phase 1: Conceptualization

An existing treatment manual for post-ACS BA-based mood management and smoking cessation was expanded to target the four health behaviors most relevant to post-ACS patients (smoking cessation, medication adherence, physical activity, and diet). This initial BA-HD treatment manual integrated guideline-driven health behavior change recommendations and mood management within a BA-framework (i.e., to develop a treatment where activation goals focus both on improving multiple health behaviors and mood management). We had already chosen our target population based on the literature discussed in the background section above. We planned to use a BA-focused approach and offer 10 visits over 12 weeks based on existing BA outcome trial literature and to initiate treatment 3–6 months after ACS. We planned for an interventionist to provide our intervention one-on-one in-person and/or by phone. We were open to any bachelor’s or master’s level providers (e.g., social workers, nurses, community health workers, master’s level mental health counselors) as interventionists at this point. However, we were particularly interested in cardiac rehabilitation professionals as potential interventionists due to their familiarity with the target population as well as recent pilot evidence that such professionals are able to facilitate a BA program [21]. We expected to provide simple, affordable tools to support behavior change and to tailor tools to each behavioral target (i.e., smoking, medication adherence, physical activity, diet). We planned these tools to be relevant to overcoming barriers to health behavior change common in people with depressed mood and to facilitate specific BA processes (e.g., goals setting, self-monitoring, stimulus control, behavioral skill acquisition). We had already chosen nicotine replacement therapy (patch and/or lozenge) as the program tool for those working on smoking cessation based on existing literature on nicotine withdrawal in those with depression, as well as the formative work our team has conducted specifically with post-ACS smokers [17, 22]. The initial conceptualization of treatment was done by AMB, WCW, and ECG.

## Phase 2: Formative qualitative research

Qualitative interviews were conducted to inform treatment development. Ten patient and eight provider interviews were conducted by two study authors (AMB, KDV). Interviews were audio recorded and transcribed. Framework Matrix Analysis was conducted using the tool in NVivo 11 to summarize and aggregate participant responses [23]. Three coders reviewed transcripts and summarized patient/provider experiences based on *a priori* and emergent domains. Saturation was assessed following each interview.

### Patient interviews methods

All human subject procedures for patient interviews were approved by the Hennepin Healthcare Institutional Review Board (HSR #17–4351) and were conducted in accordance with relevant guidelines and regulations. All participants provided written informed consent. Semi-structured interviews were conducted with ACS patients who were 3–6 months post-event with depression and who reported difficulty with ≥2 health behaviors upon screening. Patients were identified via medical record review of cardiology patients treated at an urban safety net hospital and recruited via phone calls and mail. Depression was defined as either a Patient Health Questionnaire-9 score (PHQ-9) ≥10 at any point post-ACS or a PHQ-9 ≥5 at any point post-ACS for those currently in counseling or medication treatment for depression. The rationale for including both of these groups was to get feedback from those currently depressed and from those who sought out post-ACS depression treatment. Difficulty with health behaviors were assessed via brief screening questions including: 1) one item about past 7-day use of cigarettes and one item about use of other tobacco products in the past month, 2) three items about taking medications as prescribed, skipping, or forgetting medications over the past month, 3) one item about days exercised for at least 30 minutes in the past week, and 4) three items about following a healthful diet, fruit and vegetable consumption, and high fat food consumption over the past week. Additional inclusion criteria were being between ages 18–75 and English language proficiency. Exclusion criteria were chart indication of significant cognitive impairment (e.g., chart-documented dementia), current exacerbation of psychosis, serious mental illness, or suicidality, and current hospice care.

Interviews were conducted in-person and were 60–90 minutes in length. A semi-structured interview guide was developed to elicit feedback on the following areas of inquiry: 1) interaction of mood and health behaviors post-ACS, and 2) feasibility and acceptability of the proposed BA-HD treatment, including the overall treatment rationale, treatment structure and options for potential health behavior tools to be provided as part of treatment. Patients were provided $30 of compensation for their time at completion.

### Patient interviews results

Patients interviewed (n = 10) were mostly men (80.0%) and 40.0% self-identified as Black or African American with a mean age of 55.7 ± 5.1 years. The sample included patients who reported STEMI (40%), N-STEMI (40%), and unstable angina (20%).

#### Interaction of depressed mood and health behaviors

The majority of patients (9 out of 10) reported bi-directional interaction of depressed mood and health behaviors post-ACS (i.e., poor mood worsening adherence with at least one health behavior and lack of compliance with at least one health behavior worsening mood). Several patients described this as a compounding cycle in which poor mood caused poor health behavior compliance, and this poor compliance further lowered mood. Multiple participants reported that in some circumstances, compliance with health behavior recommendations *worsened* their mood. Examples included 1) having a separate heart healthy meal from family increased feelings of isolation and dependence on others, and 2) avoiding eating salty/sugary foods or smoking led to low mood/feelings of missing out on enjoyable activities. Multiple participants also reported that attempting to make structured health behavior changes can enhance mood if successful (e.g., sense of pride/accomplishment), but can also lower mood if unsuccessful (e.g., sense of failure).

#### Reaction to the proposed treatment

Participants were presented with the treatment rationale and major treatment components. They were generally interested in the program and supportive of the BA thesis and the rationale for combining mood and health behavior intervention. Participants particularly appreciated 1) that behavior change goals would be linked to individualized values, 2) the collaborative nature of goal setting, 3) that goals would be individualized to the patient’s context, and 4) that goals would be concrete and measurable.

Preferences for format were mixed. Some strongly preferred phone sessions, some strongly preferred in-person sessions, and some preferred a mix. Patients clearly preferred sequential to simultaneous health behavior targeting. Patients who had attended cardiac rehabilitation had positive regard for rehabilitation staff and described a positive connection built during their sessions.

#### Reaction to tools

We presented participants with multiple options for tools to aid changes in medication adherence (pill boxes of different sizes and with various features), physical activity (wearable activity trackers), and diet (portion plate and a book on how to choose healthy food at restaurants). Only participants who had struggled with a given health behavior post-ACS were asked about tools for that health behavior.

Patients generally found the concept of receiving small tools to facilitate change to be highly acceptable. The most patient-desired tool for physical activity was a wearable device with a step counting feature. Patients reported this would be very useful for self-monitoring and goal setting. Some preferred a low-tech pedometer, while others preferred a device that could be paired with a smartphone. Patients had a negative reaction to the usefulness of a portion plate and were mixed regarding the usefulness of the book about healthy eating in restaurants as a useful diet tool. Multiple participants reported that a more useful tool for dietary change would be a cookbook with healthy recipes that are not time-intensive. Some participants requested similar information provided digitally. Patients were open to using a pill box to facilitate medication adherence, but preferences for features (e.g., size, number of slots, portability, alarm) were very nuanced and varied with regimen complexity. One Black male participant noted he preferred a solution that allowed him to carry the pill bottle provided by the pharmacy in case he was stopped by police and needed to provide proof of a prescription.

#### Emergent themes

About half of patients reported some stigma regarding psychiatric labels (e.g., “depression”), treatments (e.g., “counseling”), and providers. Overall, patients indicated that a program that deemphasized links to psychiatry would be more acceptable.

Interviewers noted that patients often struggled with more health behaviors than were identified by the simple, pre-interview screening questions. This was particularly true for medication adherence (i.e., multiple participants indicated during the interview that they struggled with medication adherence, but this was not caught by screening questions).

### Provider interviews method

Based on the results of patient interviews, we made the decision to utilize cardiac rehabilitation professionals (often exercise physiologists, nurses, or occupational therapists) as interventionists for our BA-HD intervention. This was based on 1) the positive experience with such providers reported by patients in qualitative interviews, 2) the high level of stigma reported regarding providers perceived as linked to psychiatry in qualitative interviews, and 3) emerging research that BA interventions can be done effectively and with fidelity by interventionists with little or no psychiatric training [13, 24, 25].

All human subject procedures for provider interviews were approved by and conducted according to the guidelines and regulations of the Hennepin Healthcare Institutional Review Board (HSR #17–4351). All provider participants provided verbal informed consent. We conducted a total of 8 semi-structured provider interviews. Six were with cardiac rehabilitation professionals (the target providers of our intervention). These included nurses (n = 2), exercise physiologists (n = 3), and an occupational therapist (n = 1). In addition, we interviewed a pharmacist (n = 1) and a dietician (n = 1) who were embedded in a cardiac rehabilitation program and worked regularly with post-ACS patients (these providers were interviewed for expertise, but were not from professions being considered as potential providers of our intervention). All providers were recruited from a large urban safety net hospital in-person or via email. All providers were women.

A semi-structured interview guide was developed to elicit feedback on the feasibility and acceptability of the proposed BA-HD treatment, including any potential health behavior tools to be provided as part of treatment. We also asked cardiac rehabilitation professionals if they would be willing to be an interventionist in a program such as the one proposed. Provider interviews were conducted in-person and were 30–60 minutes in length.

### Provider interview results

#### Reaction to the proposed treatment

Targeting depression and health behavior change in one intervention post-ACS was generally supported by all providers interviewed. Providers were generally supportive of the applicability of BA techniques. They especially liked the focus on "improving by action", using pleasurable activities to create a “positive feedback cycle”, and alignment of BA goals with patient values.

In general, providers reported that current clinical resources are often inflexible, and the BA-HD intervention should be as flexible as possible to maximize access to care in depressed ACS patients. Providers specifically reported that offering access by phone was important.

Providers had a mixed reaction to the proposed timing of intervention. Some liked the delayed timing of the intervention noting the period right after ACS is often very busy with medical appointments. Some suggested it could be offered sooner after ACS. The majority of targeted providers reported they would be willing to be an interventionist in our proposed program, but some expressed that they feel undertrained for the level of psychopathology they encounter and requested more training before program implementation. One provider reported a low level of interest in having a major role in addressing depressed mood or psychiatric issues and reported she would not be willing to be an interventionist in such a program.

#### Reaction to tools

We presented providers with multiple options for tools to aid changes in medication adherence, physical activity, and diet. The pharmacist interviewed only commented on medication adherence tools. The dietitian interviewed only commented on diet tools.

Providers reported that many patients will have a pill box already. Providers suggested that the right pill box option needs to be matched to each participant’s idiographic barriers to adherence and the complexity of medication regimen. One provider suggested ultra-portable options (e.g., necklace) for emergency medication. Providers were supportive regarding the usefulness of step-counting devices for increasing physical activity. Providers reported it is important to provide a “low-tech” version for most ACS patients. Providers had mixed opinions regarding the usefulness of a portion plate and the book about healthy eating in restaurants. Providers suggested measuring cups/spoons to aid healthy cooking.

#### Emergent themes

Basic needs, including housing, transportation, food insecurity, and lack of health insurance, contribute to depression and are major barriers to health behavior change in post-ACS patients. Some providers expressed that their efforts to assist patients with these challenges were insufficient to fully overcome these barriers.

## Phase 3: Expert panel

### Expert panel methods

Following formative qualitative research, an expert panel was convened to provide expert guidance on the planned intervention. Panel members were doctoral level clinician-researchers with expertise in relevant theory, relevant health behaviors, and cardiac psychology. The panel met for a 2-hour group meeting held during the annual meeting of a professional organization. The meeting was facilitated by KDV. Members were invited to provide feedback on planned intervention structure, content, and delivery and to generate ideas related to how to integrate planned behavior change tools with the BA goal setting process.

### Expert panel results

Expert feedback informed many details of the BA-HD manual tested in Phase 4. Expert feedback included the following: 1) Experts were supportive of cardiac rehabilitation professionals as interventionists, but noted that they would need training and ongoing support; 2) Experts were supportive of the use of step counting devices as primary tools to promote walking, but also suggested additional video options to promote exercise at home when walking may not be feasible (e.g., when there is no safe place to walk) or the patient has mobility restrictions (specifically, they suggested a curated library of low impact workout videos that could be accessed online or on DVD); 3) Experts recommended flexibility in provision of heart healthy cookbooks and recipes, specifically they recommended that interventionists personalize what is offered based on patient factors (e.g., comorbidities, cultural food practices); 4) Experts suggested that later sessions are shortened and that some patients will not need all 10 sessions planned; 5) Experts suggested the interventionists have community resource lists for resources in the community that will support goals (e.g., like safe places to walk in the winter) and/or support basic needs (e.g., list of local food pantries).

### Summary findings from Phase 1–3

Overall, feedback from the patient and provider interviews and the expert panel reinforced some aspects of the initial treatment conceptualization and also informed decisions for other manual refinements. See Table 2 for a summary of treatment development decisions.

**Table 2:** Treatment development decisions made and the source of qualitative data that informed those decisions.

	Source of data driving choices		
Treatment development decisions made	Patient	Provider	Expert
Targeting mood and health behavior in integrated treatment reinforced	X	X	
Use of Behavioral Activation as treatment modality reinforced	X	X	
Chose Sequential Targeting of health behaviors	X	X	
Chose Cardiac rehabilitation professionals as interventionists with significant training and supervision	X	X	X
Will use term “coach” as label for interventionist to reduce issues with psychiatry related stigma	X	X	
Added flexibility in treatment logistics (in-person and phone offered; flexible # of sessions)	X	X	X
Added flexibility in target population inclusion criteria*	X	X	X
Changes to behavior change tools provided**	X	X	X
Developed a community resource library available to make available to providers		X	X

*See phase 4 inclusion/exclusion in section 4.2 below **See *Summary of BA-HD intervention* for final tools offered in Phase 4.

## Phase 4: Pilot testing

Pilot testing consisted of two demonstration cases with coaching conducted by a clinical psychologist (AMB) followed by an open trial with coaching conducted by a trained cardiac rehabilitation professional. Recruitment and inclusion/exclusion criteria were the same for demonstration cases and the open trial. All human subject procedures in this phase were approved by the Hennepin Healthcare Institutional Review Board (HSR #17–4351). All procedures were conducted in accordance with the institutional review board guidelines and regulations. All participants provided written informed consent. The study was registered with Clinicaltrials.gov (NCT04158219).

The recruitment process started with a review of all cardiac catheterization cases conducted from January 2018 to September 2020 at an urban safety net hospital. These cases were reviewed by a research staff member to identify all catheterizations that resulted in an ACS diagnosis. Outreach letters were sent to ACS cases who met age and language criteria followed by outreach phone calls. These letters and calls invited patients to be screened by phone using the criteria below. Patients who completed and met criteria from the phone screener were invited to enroll.

Inclusion criteria were: 1) ACS occurrence within the past 2–12 months, 2) post ACS depression based on either post-ACS depression documented in the medical record (i.e., documentation of a formal depression diagnosis post-ACS, a self-report scale score in chart indicating likely clinical depression post-ACS) or a 10-item Center for Epidemiological Studies Depression scale (CES-D-10) score ≥ 10 upon screening, 3) non-adherence to and willingness to implement changes to ≥1 behavioral risk factor, 4) age 18–75, and 5) English language fluency. Exclusion criteria were chart indication of significant cognitive impairment (e.g., chart-documented dementia), current exacerbation of psychosis, serious mental illness, or suicidality, and current hospice care. Patients were also excluded if currently engaged in cardiac rehabilitation or other regular counseling treatment targeting depression or health behavior change.

### Demonstration cases

The first two participants enrolled were coached by AMB (a licensed clinical psychologist) in order to demonstrate implementation of the BA-HD treatment manual. The primary purpose of these sessions was to provide example audio, clinical notes, and BA goals to supplement the treatment manual and inform the subsequent training of cardiac rehabilitation professionals as interventionists. These cases also served to finalize forms and procedures so that these were standardized before training other coaches. One demonstration participant worked on smoking and attended eight sessions (2 in-person in clinic and 6 sessions via telephone). One participant attended one in-person session and planned to work on diet, but he had to end participation due increased professional responsibilities during the COVID-19 pandemic.

### Open trial method

An open trial with coaching by a trained cardiac rehabilitation professional (JKC) was conducted to assess treatment feasibility and acceptability. Following enrollment, each participant completed a baseline assessment. An end-of-treatment assessment and exit interview were conducted 12 weeks later regardless of how many sessions were completed. These visits were planned to be in-person either at the clinic or the individual’s home, but due to COVID-19 restrictions, phone assessments were utilized to minimize in-person contact. Participants were compensated $30 for the baseline assessment and $50 for the end-of-treatment assessment and exit interview.

#### Summary of the BA-HD intervention

BA-HD was designed to target both depressed mood and four health behaviors (smoking, medication adherence, physical activity, and healthy eating) post-ACS. The BA-HD intervention manual was informed by existing BA for depression manuals (particularly Kanter, Busch, Rusch, 2009 [12]), an existing manual integrating BA with smoking cessation post ACS [17], American Heart Association recommendations for health behavior change, and feedback from phases 1–3. The BA-HD manual was specifically designed to be provided by cardiac rehabilitation professionals or other individuals with general medical knowledge, but without specific training in behavioral health. Per feedback from phase 2, the interventionist was referred to as a “health coach”.

The BA-HD treatment consisted of eight to ten sessions of individual coaching over twelve weeks. The original plan was for the initial two sessions (50 minutes each) to be conducted in-person (in clinic or in the patient’s home). However, following initiation of the COVID-19 related stay at home order, sessions 1 and 2 were completed by phone. Sessions 3–10 (30 minutes each) were all completed by phone as originally planned.

Session 1 focused on acclimating the patient to the coaching program. After introductions, the health coach learned more about the patient’s history with depression and their cardiac event. Then, the coach explained the rationale for BA-HD (e.g., setting active goals to improve mood and decrease cardiovascular risk). The coach and patient worked together to identify a health behavior to target and self-monitor. If there was time, the coach would assist the patient in setting one small goal.

Session 2 started by reviewing the interactions between mood and activity over the past week (using the self-monitoring sheet from session 1). If a goal had been set in session 1, the two would review the successes and barriers the patient encountered over the week. The bulk of session 2 focused on a structured values assessment which was then used to set mood-enhancing, value-consistent goals (i.e., goals that were pleasurable and/or values-driven). During session 2, after deeper exploration of values, participants set four small goals for the upcoming week.

To support behavior change, cost-effective aids were offered relevant to each targeted health behavior. During the COVID-19 pandemic, tools were either mailed to or directly dropped off at patient homes with instructions. For each health behavior, the coach addressed how the health behavior relates to mood and cardiac health. Then, the coach and participant worked collaboratively together to choose a tool to help achieve set goals. The coach provided instruction on usage of the tool and how to track changes in health behaviors and goals.

For those focused on smoking, nicotine replacement therapy (lozenges or patches) were offered. The coach offered individualized pill carrying, reminder, and organization tools to participants addressing medication adherence. For goals based around physical activity, patients were offered instructive videos, simple electronic pedometers, or a wearable accelerometer with more features (e.g., linking to smartphone, heart rate monitoring). Participants worked collaboratively with their coach to find tools that were the most relevant. For diet, participants could receive measuring cups and nesting bowls as well as cookbooks and recipes that were individualized (based on Phase 2 feedback) to the patient’s specific goals and comorbidities.

Sessions 3–10 were done via phone (both pre and post COVID-19) and reviewed health behaviors, mood, and activity, with an emphasis on reinforcing value-behavior-mood associations and setting new activation goals. As goals were met, the participant and coach worked collaboratively to decide whether to set new goals or to step up (in intensity, duration, or frequency) previous goals. Throughout treatment, participants were offered text or email reminders about their goals for the week. At the fourth session, participants were able to start setting goals for a second health behavior if relevant and desired. In final sessions the coach focused on how the patient would continue progress after the end of treatment.

#### Interventionist and interventionist training and supervision

Intervention sessions were delivered by an exercise physiologist whose primary role is providing cardiac rehabilitation care. This coach was trained through 1) reading the BA-HD treatment manual and didactic review of the manual with AMB, 2) background readings on Behavioral Activation for depression (e.g., Kanter, Busch, and Rusch 2009 [12]), 3) review of demonstration case session recordings and notes, and 4) role play of each session in the manual followed by feedback from AMB. Throughout the open trial, AMB listened to sessions regularly and met with the coach weekly to provide feedback and consult on cases.

#### Measures

Assessments were completed in-person or telephone at baseline and end-of-treatment assessments. Assessments were conducted by a research staff member (not the coach). Age, gender, ethnicity, marital status, education background, employment, income, medical co-morbidities and mental health treatment history were self-reported. Acceptability and treatment engagement were identified as the primary outcomes *a priori*.

*Acceptability*. Treatment acceptability was assessed with the 8-item Client Satisfaction Questionnaire [26]. Scores range from 8 to 32, with higher scores indicating greater satisfaction with treatment quality, quantity, and procedures.

*Treatment engagement*. Number of sessions attended and treatment completion were used to assess treatment engagement; treatment completion was considered attendance of at least 8 sessions.

*Retention*. Study retention was defined as the percentage of participants who complete the end-of-treatment outcome assessment.

*Depressive symptoms*. The PHQ-9 and CES-D-10 were used to measure depressive symptoms. The PHQ-9 [27] measures self-reported depressive symptoms via nine items that assess depressive symptom severity over the past two weeks. Scores range from 0 to 27, with scores ≥10 indicative of at least moderate depressive symptom severity. The 10-item CES-D-10 [28] was also administered as a measure of self-reported depressive symptoms over the past week. Scores range from 0 to 30 with higher scores indicative of greater symptom severity.

*Composite behavioral risk factor adherence*. 10 items from the Medical Outcomes Study Specific Adherence Scale [29] were used as a composite indicator of the targeted behaviors. These 10 items have been identified as relevant to cardiac patients and used in previous studies with post ACS patients (e.g., Ziegelstien et al. 2000 [30]; Romanelli et al. 2002 [31]). Each item asks participants to rate frequency of behavioral engagement relevant to diet, exercise, medication adherence and disease self-management, tobacco cessation, stress management, and utilization of social support over the past four weeks. Each item is rated as 1 (“none of the time”) to 6 (“all of the time”). Some of the 10 items were not applicable to all participants (e.g., one item asks about following a diabetic diet). A total score was calculated for each participant that averaged the responses to individually-relevant items.

*Smoking Cessation*. The average number of cigarettes smoked per day over the last week was self-reported.

*Medication adherence*. Medication adherence was assessed using 3 self-report items from the Heart and Soul and CARDIA studies [32, 33]. One item assesses the frequency of taking medications as prescribed over the past month (response options range from “all of the time [100%]” to “less than half the time [<50%]”). The other two items assess forgetting to take ≥ 1 medication and intentionally skipping ≥ 1 medication in the past month (response options range from “never” to “nearly every day”.

*Exercise*. One item queried the number of days in the past week that the participant engaged in ≥ 30 total minutes of exercise (i.e., physical activity that was not part of the participant’s employment and intense enough to elevate an individual’s breathing rate; [34]).

*Diet*. Dietary behavior was assessed using three items from the Summary of Diabetes Self-Care Activities measure [35] that queried number of days in the past week following a healthful eating plan, consuming ≥5 servings of fruits and vegetables, and eating high fat foods. These items are reported separately.

*Positive affect*. The positive affect subscale of the Positive and Negative Affect Scales- Short Form (PANAS-SF [36]) measured individual ratings of experience of 10 emotions during the past week on a 1 (“very slightly or not at all”) to 5 (“extremely”) scale. For the 5-item positive affect subscale, scores range from 5 to 25, with high scores indicating higher positive affect. We measured positive affect because it is a strong and independent predictor of post-ACS morbidity and mortality.

Participants also completed an informal qualitative exit interview regarding study logistics and the structure and content of the intervention. All interviews were conducted by a research staff member not involved in the participant’s treatment.

#### Analytic plan

Means, standard deviations, frequencies, and percentages were calculated to describe the sample. Because the intent of the open pilot trial was feasibility and acceptability assessment to inform additional treatment refinement, analysis emphasized descriptive characterizations of the data and calculation of effect sizes to examine clinical magnitude of preliminary effects. Descriptive data on baseline characteristics and treatment engagement are presented on the entire sample, regardless of trial completion. Treatment satisfaction and pre- to post- change were calculated only for completers. Analyses were performed in Stata SE 16.

### Open trial results

#### Sample characteristics

**Fig 1:**
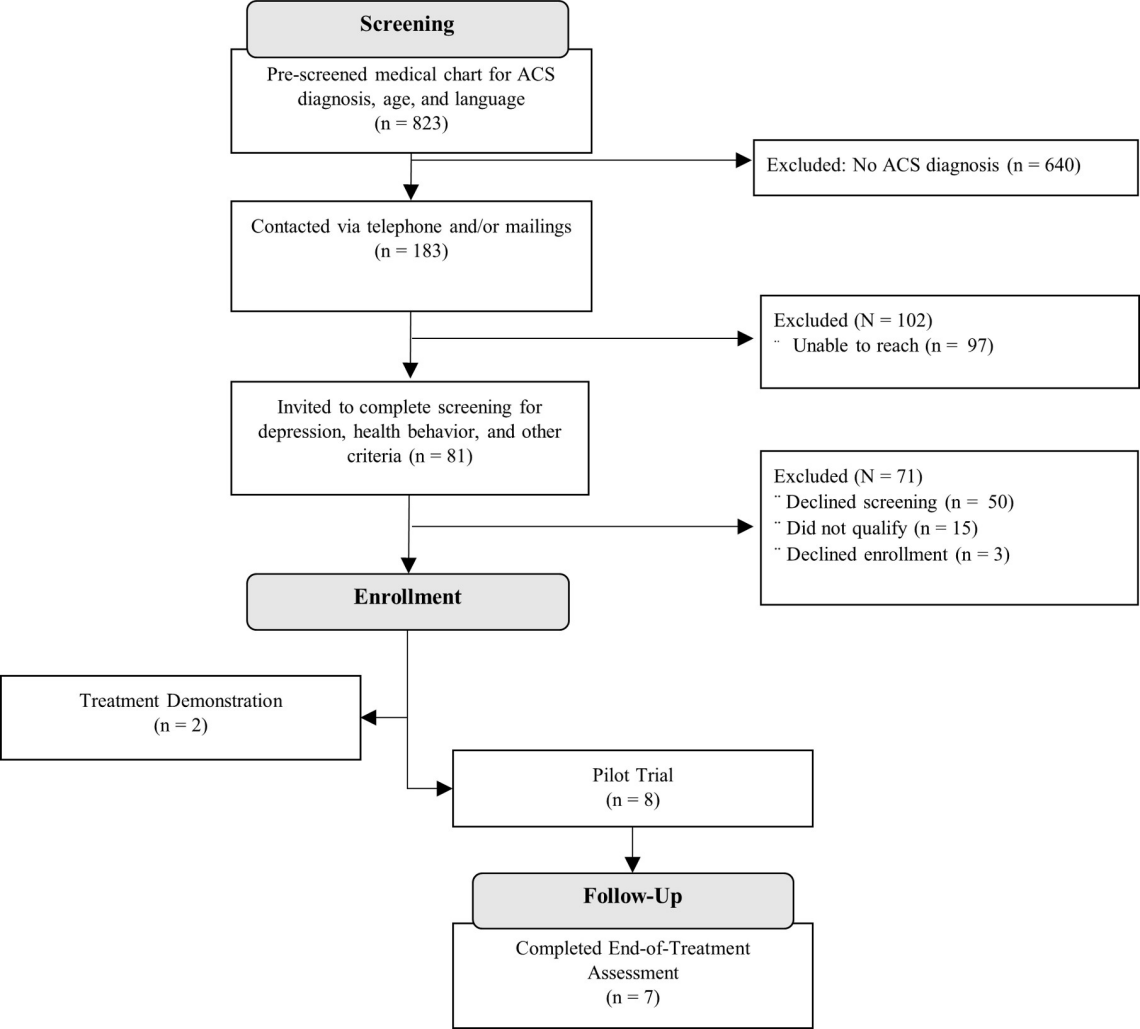
Participant selection and study flow.

The flow of participants through all procedures can be seen in the Consort Diagram (Fig 1). Although the planned target was 20 participants, eight participants were enrolled in the pilot trial due to timing challenges related to COVID-19 and funding. Enrolled participants mostly identified as non-Hispanic White men (see Table 3). Four participants had a diagnosis of STEMI, two had a diagnosis of NSTEMI, and the other two had a diagnosis of unstable angina. Half of the participants were married or living with a partner. Six out of the eight trial participants reported a history of mental health treatment.

**Table 3:** Open pilot trial participant characteristics at baseline (n = 8).

Demographic Characteristics	Mean (SD) or n (%)
Age	57.88 (13.01)
Women	3 (37.5%)
Race and ethnicity	
Black or African American	3 (37.5%)
White non-Hispanic	4 (50.0%)
White Hispanic	1 (12.5%)
Marital Status	
Divorced	2 (25%)
Married/living with a partner/in a committed relationships	4 (50%)
Never married	2 (25%)
Highest education level completed	
Grades 9–11	1 (12.5%)
High School	3 (37.5%)
Some college	2 (25%)
College graduate	2 (25%)
Employment status	
Employed full-time	1 (12.5%)
Employed part-time	2 (25%)
Retired	2 (25%)
Disability	3 (37.5%)
**Medical Comorbidities**	
Type 2 Diabetes	4 (50%)
Congestive heart failure	3 (37.5%)
Peripheral artery disease	2 (25%)
Chronic obstructive pulmonary disease	1 (12.5%)
Chronic kidney disease	1 (12.5%)
Stroke	0
**Mental Health Treatment**	6 (75%)

#### Primary outcomes: Acceptability and treatment engagement

The median number of sessions completed was 10 (range: 2 to 10). Seven out of eight (88%) completed at least 8 sessions and five out of eight (63%) completed all 10 intervention sessions offered. Of note, the first participant enrolled attended the first two sessions in-person at the clinic. All subsequent participants could only be offered phone sessions due to the implementation of COVID-19 stay-at-home orders. The mean CSQ score was 30.57 ± 2.23, indicating high satisfaction.There were no adverse events during the trial.

Seven exit interviews were conducted. Participants generally reported that the coaching was helpful. In particular, participants noted that the coach and the values based, collaborative goal-setting approach was helpful. Five participants expressed finding the focus on mood and behavior interactions to be beneficial, and some noted increasingly recognizing their own patterns of mood-behavior cycles. Five participants specifically offered that receiving text message or email reminders of goals set in coaching was helpful. All participants found the telephone sessions to be acceptable. All participants reported that the duration and number of sessions were adequate; two expressed that they would have been open to more sessions.

#### Secondary outcomes: Retention, depressive symptoms, and health behavior change

*Feasibility of recruitment*. Of 31 patients who agreed to screening procedures, 15 were ineligible following screening, 3 were not interested, and 3 expressed interest, but did not complete baseline procedures. Two were enrolled as demonstration cases and 8 were enrolled in the open trial.

*Retention*. Seven out of eight pilot trial participants completed the end-of-treatment assessment, including the exit interview. See Fig 1 for details on recruitment and retention.

*Depressive symptoms*. Improvements in PHQ-9 and CES-D scores were consistent with a medium effect size (Table 4).

**Table 4:** Mood, affect, and health behavior at baseline and end-of-treatment for completers (n = 7).

	Baseline (M ± SD)	End-of-Treatment (M ± SD)	*d* ^*a*^
**Depressive Symptoms**			
CES-D-10	11.43 ± 4.72	8.57 ± 4.54	0.62
PHQ-9	8.29 ± 5.12	4.86 ± 3.89	0.75
**Health Behavior**			
MOS Average Score^b^	3.75 ± .94^c^	4.53 ±0.70^d^	.94
**Affect (PANAS)**			
Positive Affect	14.29 ± 3.64	16.71 ± 2.36	0.79

^*a*^*ds* were calculated such that a positive *d* value represents improvement.

^***b***^ *n*’s vary according to number of relevant items for each participant.

^c^ *M* # of items: 7.42 ± .98.

^d^*M* # of items: 7.86 ± .90.

*Composite behavioral risk factor adherence*. The average MOS score improved from pre- to post-test and was suggestive of a large effect size (Table 4).

*Diet*. Six participants worked on elements of dietary change such as increasing fruit and vegetable consumption, portion control, and improving diet quality by cooking at home with heart healthy ingredients. Participants who targeted diet reported an increase following a healthful eating plan from a mean of 3.17 (*SD* = 2.79) to 6 (*SD* = 1.26) days per week. Eating recommended servings of fruits/vegetables increased from a mean of 2.67 (*SD* = 2.66) to 5.5 (*SD* = 2.81) days per week. There was no change in days eating high fat foods (Baseline: *M* = 2, *SD* = 1.41; End-of-treatment: *M* = 2.33, *SD* = 2.58).

*Exercise*. Five participants worked on increasing physical activity. The average number of days exercised ≥30 minutes increased from 1.4 (*SD* = 1.14) days to 4.4 (*SD* = 1.52) days per week.

*Smoking cessation*. Three participants worked on smoking cessation. One of these participants dropped out of treatment after two sessions. One individual reported smoking 5 cigarettes a day at baseline and quit smoking by end-of-treatment. The other participant reduced daily cigarette use from 10 cigarettes per day at baseline to 4 cigarettes per day at end-of-treatment.

*Medication adherence*. Two participants worked on medication adherence. One participant reported improvements in forgetting medications (“once per week” to “once in the past month”) and skipping medications (“once in the past month” to “never”). One participant focused on medication adherence as a secondary target and did not report any improvement.

Notably, the two participants who worked to improve medication adherence did not exclusively work on medication frequency, but rather often focused on other elements that supported adherence such as refrigerating medications appropriately, carrying emergency medications when outside of the home, and improving the temporal consistency of medications taking (which ultimately improved side effects).

## Discussion

An integrated mood management and health behavior treatment was iteratively developed through refinements based on formative qualitative work and expert feedback. This culminated in a single-arm pilot trial of the BA-HD treatment. The trial enrolled eight participants and demonstrated high treatment satisfaction, treatment engagement, and study retention. Seven out of eight participants completed treatment and the end-of-treatment assessment. Although findings should be considered preliminary in light of the pilot nature of this work, treatment led to meaningful changes in the expected direction for depressive symptoms, positive affect, and relevant health behaviors. Our findings are generally consistent with prior work supporting the integration of behavioral activation-based approaches targeting depression in medical settings and targeting health behavior [17, 37, 38]. Trial participants reported that BA-HD reinforced their understanding of mood-health behavior relationships and they appreciated the values-guided and collaborative nature of goal-setting. BA based approaches for mood and health behavior change post-ACS warrant continued treatment development and testing.

We wrote the BA-HD manual to be conducted by bachelors-level cardiac rehabilitation professionals, and a current cardiac rehabilitation professional (JKC) served successfully as the coach conducting BA-HD in the open trial. We made this decision based on our qualitative findings as well as a growing literature indicating that individuals with no special training in mental health counseling can conduct BA with fidelity with minimal training. The use of cardiac rehabilitation professionals as coaches has significant advantages over relying on mental health providers (e.g., psychologists, licensed social workers). First, it may avoid some of the stigma associated with psychiatric care reported by patients in phase one qualitative interviews. Second, cardiac rehabilitation professionals have deep knowledge of relevant post-ACS health and relevant behavior change targets, so only limited BA related training and supervision are needed. Finally, if BA-HD proves to be effective in larger trials, dissemination could be aided by the lower cost of cardiac rehabilitation professionals. Notably, the American Heart Association’s stated goals of an optimal Phase II cardiac rehabilitation program include addressing the post-ACS patient’s physical, psychological, and social health via multifaceted approaches [39]. In line with these aims, BA-HD dissemination efforts could leverage existing cardiac rehabilitation centers as hubs for provision of BA-HD.

Several observations from the open trial revealed areas for additional refinement of study procedures and assessment protocols. First, of 183 ACS patients with ACS that we attempted to contact by phone and mail, only 81 were successfully contacted, and only 31 agreed to screening. Much of study recruitment took place during the COVID-19 pandemic which may have affected willingness to be screened and participate. It is clear that there were fewer ACS cases seen in US hospitals early in the COVID-19 pandemic, which likely reduced the overall pool of eligible participants. Regardless, future recruitment efforts will likely require different strategies such as in-person recruitment and multiple recruitment sites.

Second, alternative approaches to assessing change in multiple health behaviors are needed. From our clinical observations and the data regarding individual health behaviors, it is clear that several open trial participants made significant progress on improving one or more health behaviors during the study. Although potentially limited by the small sample size, our composite outcome measure (the MOS) did not capture this improvement well. While the ten MOS items used as a health behavior composite in this study have been used successfully at a single time point in cardiac patients before [30, 31], to our knowledge they have not been used as a longitudinal outcome measure. Future work should explore a different composite or more extensive measurement of individual health behaviors to more fully capture treatment-related change. Future work may also consider multimodal assessment approaches that incorporate health behavior engagement measures such as pill counts or other forms of electronic behavior monitoring in addition to self-report.

Limitations that impact the interpretation of our pilot trial findings include the small sample size, use of a single site and interventionist, inclusion of self-reported adherence only, and unknown impact of COVID-19 on preferences surrounding treatment delivery. Although encouraging improvements in mood and behavior were observed, interpretations of change and effect sizes should be interpreted in light of the pilot nature of this trial.

## Conclusions

This paper describes the multi-phase process undertaken to develop and refine an integrated mood management and health behavior counseling treatment for ACS patients with depression and poor health behavior adherence (BA-HD). Through phases of conceptualization, formative qualitative research, and expert feedback, we developed and refined the BA-HD manual. Importantly, pilot testing demonstrated the feasibility and acceptability to patients of targeting depressed mood within the context of behavioral cardiac risk reduction efforts and the incorporation of individualized personal core values into health behavior change efforts. Planning for a larger trial is underway to further determine the efficacy of BA-HD to benefit mood and health behavior targets.

## Supporting information

S1 File Pilot test protocol. (DOCX)

S2 File TREND checklist for the pilot test. (DOCX)
