# Supplementary material for: The development and pilot testing of a behavioral activation-based treatment for depressed mood and multiple health behavior change in patients with recent acute coronary syndrome
Source: PLoS One. 2022 Feb 3;17(2):e0261490. doi: 10.1371/journal.pone.0261490 (PMC8812840; doi:10.1371/journal.pone.0261490)
Supplement: S1 File — (DOCX) [file pone.0261490.s001.docx]

# HSR #17-4351: Development of an Integrated Depression and Behavioral Risk Factor Reduction Intervention for Secondary Prevention Following Acute Coronary Syndrome

# Approach

# Aim II: Conduct an open trial (N=20) of BA-HD to test the feasibility and acceptability of our procedures and obtain initial indications of efficacy.

Inclusion criteria: 1) ACS diagnosis (diagnosis of unstable angina, ST and non-ST elevation myocardial infarction) documented in medical record in the preceding 2-12 months, 2) Depression diagnosis listed on patient problem list in patient’s medical record, a clinically administered PHQ-9 score of 10 or greater in the past 12 months indicated in the patient’s medical record, or a Center for Epidemiologic Studies Depression scale (CESD) score ≥10 (indicating “likely major depressive disorder”); CESD will be administered by phone during screening if there is no indication of depression or PHQ-9 score within 12 months in the patient medical record, 3) current non-adherence to ≥ 1 of four behavioral risk factors (see below), 4) willing to make immediate changes to ≥ 1 relevant behavioral risk factors, 5) age of 18-75, 6) lives within 1.5 hours of Hennepin Healthcare, and 7) fluent in English.. Exclusion criteria: 1) Limited mental competency (as indicated in medical chart), 2) presence of current exacerbation of psychosis/serious mental illness or suicidality, 3) in hospice care, 4) currently attending regular counseling targeting depression or any health behavior change, and 5) currently attending a cardiac rehabilitation program (those excluded for being in rehabilitation will be re- contacted after completion). We will attempt to obtain a sample of at least 45% women, which is representative of the gender distribution of discharged ACS patients.^1^

We will define non-adherence as follows: Tobacco non-adherence = use of any tobacco product in the last week. Physical activity non-adherence = engagement in < 90 minutes of moderate or greater physical activity per week.^104^ Medication non-adherence = non-adherence based on Heart and Soul study criteria i.e., taking medication as prescribed 75% of the time or less, forgetting medications at least 1x/week, or skipping a medication at least 1x/week. Diet non-adherence = following a “healthful eating plan” 4 or fewer days per week, eating 5 or more servings of fruits and vegetables 4 or fewer days per week, or eating high fat foods 3 or more days per week.

Recruitment: Participants will again be recruited through review of electronic medical records at Hennepin Healthcare. We will pull records of recent ACS hospital discharges, screening for depression where available. The ACE team may identify names from EPIC to facilitate this. We will also review records of outpatient cardiology (clinic and cardiac rehab to identify potential patients, (a flyer will be provided to patients and providers. Flyer attached). Opt out letters will be sent to all participants identified through chart review and a research assistant will call patients on this list to screen for eligibility using the above criteria. Those that qualify by phone screen will be invited to an in person meeting to provide informed consent and then complete a baseline assessment. If study staff are unable to reach potentially qualifying patients by phone, study staff will work with care providers in clinic or cardiac rehab to introduce the study during a clinical visit, where research assistant will screen in person. The research assistant will then schedule the patient for a first session of BA-HD coaching at the Berman center, other Hennepin Healthcare facility, or in patient’s home when needed; (Note that “Home” can be a nursing home, assisted living, rehab center, or other institution.). We may recontact patients who have completed primary outcome in other Busch Lab studies.

# 4.2a Behavioral Activation for Health and Depression (BA-HD)

Consistent with successful BA manuals, we plan to conduct up to 10 sessions of treatment over 12 weeks (the recommendation will be to do at least 8 sessions; scheduling of sessions will be flexible and conform to patient preference). The initial two sessions will be about 50 minutes long and later sessions will be 20-30 minutes long. Sessions can be done on site or over the phone. The recommended treatment will have 2 sessions in person followed by phone sessions. Home visits will be offered for sessions 1-2 if a participant cannot travel to on-site sessions. Study treatment will be conducted by an exercise physiologist who is a cardiac rehabilitation provider, a HHRI employed licensed social worker, or a licensed clinical health psychologist with clinical privileges at Hennepin Healthcare.

BA has its underpinnings in the behavioral model of depression,^75^ which purports that depression results from reduced engagement in positively reinforcing activities. According to BA theory, depression and related problem behaviors can be reduced by reconnecting patients with healthy sources of positive reinforcement. The core components of all BA treatments are 1) Assessment of participant values, 2) self- monitoring of mood and behavior between sessions, and 3) Between session goal setting. We will integrate behavioral risk factor improvement into these core components.

Assessment of participant values is accomplished through a structured values assessment. The results of this assessment will be used to guide content of goals for increasing pleasant/valued activities in order to improve depression. Personal values will also be used to maintain motivation for behavioral risk factor changes. Patents will be asked to self-monitor mood and behavior between sessions in order to gather idiographic data on the relationship between mood and daily activities. Participants will also monitor the behavioral risk factors currently being targeted (see below). This will allow for discussion of the relationship between mood and behavioral risk factors. Participants will be provided with standard BA self-monitoring forms, with added sections for tracking behavioral risk factors. Between session goal setting is accomplished though collaboratively defined, between-session “activation goals” (i.e., explicitly scheduled between session activities). Goals are pleasurable and/or value driven, but specific content is individualized. Several aspects of BA facilitate the completion of activation goals. Specifically, goals are collaboratively determined, set in manageable increments (to increase self-efficacy through early success), and reviewed in detail during subsequent sessions in order to identify and problem-solve any barriers encountered.[^75^](#_bookmark36)[^,107^](#_bookmark44) In addition, all activation goals are explicitly written down (e.g., to-do list, daily planner) or otherwise scheduled (e.g., entered into cell phone calendar). With the participant’s permission, study staff will send an automated email or text message between sessions to remind the patient to complete their goals (in our pilot RCT (see section 3.2)

>80% of participants were willing and able to receive goal reminders by text or email). There is emerging evidence that such text messages can improve post-ACS adherence.^108^ Texting and emailing are included in the informed consent document. Further, when obtaining contact information, we will confirm with patients what email address we can use and what phone numbers they give us permission to text message to.

Behavioral risk factor change goals will be set alongside depression focused goals (see below) and the content of goals will be integrated to maximize both mood improvement and risk factor change. For example, for a participant trying to quit smoking, the interventionist will ensure that goals don’t involve increased contact with smoking triggers. Likewise, the interventionist will encourage pleasant/valued activity content that is consistent with risk factor targets (e.g., walking, cooking healthy a meal with spouse) and discourage content inconsistent with risk factor goals (e.g., outings for ice cream).

Risk factors will be targeted sequentially. First, the interventionist and participant will collaboratively choose the first risk factor to target. This will primarily be based on what the participant is most motivated to change. If the participant is equally motivated to change two or more risk factors on which they are non- adherent, the priority order will be: 1) tobacco, 2) medication adherence, 3) physical activity, 4) diet. This priority order is based on strength of data implicating each as a mechanism of the depression to post-ACS mortality relationship. [^14-17^](#_bookmark13)[^,76-79^](#_bookmark37) Next, the interventionist will provide brief education regarding the importance of that risk factor post-ACS, the recommended level of that risk factor for secondary prevention, and how their current behavior compares to recommendations. This information will be based on the latest clinical guidelines.**^109-111^** The interventionist will then collaboratively set an attainable goal regarding that risk factor until the next session and ask the participant to self-monitor engagement in the risk factor. A second and/or third risk factor could be targeted later in treatment if the participant makes sufficient progress on the first. The detailed content of coaching for each of the behavioral risk factors has been developed by the investigative team and is informed by clinical guidelines and our Aim I qualitative findings.

We will provide relevant educational content to support health behavior change. Content will be produced by, or closely adapted from the American Heart Association, and in-line with current guideline-based care (patients will receive a packet with this information). The behavioral targets for improving health behavioral will be as follows: **1)** For tobacco use, we will focus on quitting tobacco completely during the study period. We will allow goals to partially reduce tobacco use, but only in the context of “cutting down to quit” (i.e., we will support tobacco reduction goals that are first steps towards a goal of quitting completely; we will not support reduction itself as a long-term goal). **2)** For physical activity, we will focus on increasing overall walking steps per day. **3)** For medication adherence, we will focus increasing days that participants are adherent to their currently prescribed medications. We will encourage participants to resolve issues (e.g., cost, side effects) in discussions with their clinical providers. **4)** For diet, we will focus on making small, concrete changes to diet, consistent with the “small changes approach”. For example, we might set goals like, “eat one more fruit per day” or “limit fast food to 2x per week”. We will not focus on weight loss as a short term goal. As we have done in our previous studies (K23HL107391, AHA 12CRP9840018), we will also provide tools to facilitate behavioral risk factor change that are cost effective and widely used in clinical care. Specifically, we will: **1)** provide courses of the nicotine patch and/or lozenge to tobacco users who are willing to make a quit attempt in the next 30 days when medically indicated. Nicotine replacement is medically indicated for the vast majority of smokers with few exceptions (e.g., pregnancy). If nicotine replacement is provided the dose will be determined in consultation with study team (i.e., Dr. Busch: psychologist/tobacco expert; Dr. Ayenew: cardiologist; Dr.

Vickery: family physician). Non-daily tobacco users will only be offered the nicotine lozenge.The patient’s primary provider will be informed through EPIC messaging that nicotine replacement was provided to the participant. The study MDs/PhD may talk to the participant to obtain more information in order to assess appropriateness of nicotine replacement. Participants will be encouraged to contact staff if they have any side effects or concerns. Providing nicotine replacement is important because recent data indicate that smokers with depression symptoms experience more nicotine withdrawal post-ACS.^112^ **2)** Provide wearable accelerometers/pedometers to those attempting to increase physical activity. Accelerometers/pedometers will facilitate physical activity monitoring and goal setting. Physical activity promotion will focus on increasing step counts on accelerometers/pedometers by walking more. It is safe for the vast majority of post-ACS patients to start a walking program. If the interventionist has any concern regarding the safety of walking Dr. Ayenew and Dr. Vickery will be available for consultation. The participant may want to work on more intense exercise, especially later in the treatment protocol. If this is the case the interventionist will discuss with the patient if they have been cleared by their medical team to engage in the desired activity. If there is any concern regarding clearance for exercise, the interventionist may contact the patient’s providers and/or consult with Dr. Ayenew and Dr. Vickery. **3)** Provide a variety of pill boxes with and without reminder alarms. We will also offer reminder devices that replace pill bottles if participants prefer. We will also encourage those targeting medication adherence to set a cell phone alarm for every daily dose. Interventionists will assist participants in setting these cell phone/pill box/pill bottle top alarms. **4)** Provide those targeting diet change with American Heart Association-supported cookbooks and/or simple measurement and healthy cooking tools to facilitate healthy cooking.

BA-HD will have the following structure: **Session 1** will be offered in our clinic or at home (based on participant need and preference). Session 1 will include the following: **1)** review patient history, **2)** review status of 4 health behavior targets, **3)** discussion of rationale for BA-HD, **4)** choice of initial behavioral risk factor target, **5)** brief education and history regarding chosen health behavior, **6)** introduction of self-monitoring of mood and behaviors (including relevant health behaviors), **7)** collaboration to set one activation goal to be completed before the next session, **8)** problem-solving of barriers to completion of goals, and **9)** offering an automated email or text message reminder of goals. Participants will be provided with a packet of educational materials to keep at session 1. If applicable, interventionist will engage providers and the study team (including study cardiologist) to assess for safety of physical activity or use of nicotine replacement.

Session 2 will be scheduled in person if possible. **Session 2** will include the following: **1)** follow-up on between session self-monitoring and any planned activities, **2)** a structured values assessment^107^ and a discussion of how resulting values relate to risk behaviors (e.g., “How will continued smoking detract from grandparenting?”), **3)** provision of tools to aid risk behavior change, **4)** collaboration to set 1-4 activation goals to work on before the next session, **5)** problem-solving of barriers to completion of goals, and **6)** offering an automated email or text message reminder of goals.

**Sessions 3-10** will be conducted by phone and will follow the same basic format: **1)** assess depressed mood, activity, and relevant behavioral risk factors between sessions, **2)** review adherence to activation goals from the previous session; **3)** choose 1-4 activation goals that are pleasant and/or in line with the participant’s values and explicitly schedule these; **4)** problem-solve barriers to completion of new goals, and **5)** offer an automated email or text message reminder of goals. Starting in session 4 the interventionist and participant will collaboratively discuss if the next relevant behavioral risk factor should be added to goals. During the final coaching call (session 10 if full protocol completed), rather than assigning new activation goals, the interventionist will guide the participant in completion of a written guide which provides a structure for continuing to work towards goals independently.

# 4.2b Aim II Measurement

Participants will complete self-report, interview, and anthropomorphic measures at enrollment and at end-of-treatment (i.e., following 12 weeks of BA-HD coaching). In addition, at end-of-treatment a 30-45 minute qualitative interview will be conducted. Participants will be compensated $30 per assessment and an extra $20 for completion of the qualitative interview. Thus, Aim II participants could receive $80 total for assessments.

Transportation will also be provided or reimbursed as well.

**Variables assessed at baseline only:** Socio-demographics, including age, gender, ethnicity, employment, and income will be collected. Medical history will be assessed through self-report and chart review and will include ACS diagnosis and treatment, and a detailed history of cardiac disease, events, and procedures. Co-Morbid conditions will also be assessed.

**Outcomes:** Acceptability will be assessed only during the end-of-treatment assessment using the 8- item Client Satisfaction Questionnaire.^94^ Feasibility will be assessed by rate of recruitment/retention, session attendance, and completion of between session goals. Depression symptoms will be assessed using the CESD and the 9-item self-report Patient Health Questionnaire (PHQ-9).^97^ Composite Behavioral risk factor adherence: The Medical Outcomes Study Patient Adherence Questionnaire^106^ has validated items measuring all four of the behavioral risk factors targeted (tobacco, physical activity, diet, medication adherence) and has been used in large studies of post-ACS risk factor change.^113^ Thus, we will use the total score of these items as a composite outcome across the 4 risk behaviors. We will calculate the total score for the 10 more general items related to cardiac health ^135^. We will also administer brief validated questions regarding time spent engaging in physical activity, medication adherence, and diet quality. Change in tobacco use will also be measured continuously with cigarettes/other tobacco product use per day and dichotomously using 7-day point prevalence abstinence (i.e., no use at all in past 7 days). A breath sample will be used to verify abstinence[.^117^](#_bookmark45)[^,118^](#_bookmark46) Participants who report using smokeless tobacco products or e-cigarettes containing nicotine will be considered tobacco users. **Secondary outcomes**: We will also include established self-report measures of affect,^119^, health related quality of life^121^, exercise capacity,^122^ and hypothesized BA mediators.^123^[^,124^](#_bookmark47) Weight/height, waist circumference, and resting systolic and diastolic blood pressure will be collected using standard procedures.

# 4.2c Analysis

Qualitative Data: We will again analyze recordings of end-of-treatment qualitative interviews using the Framework Matrix Analysis procedures described for Aim I above. However, analysis of follow-up interviews will explicitly focus on acceptability and suggestions for improving BA-HD.

Feasibility and Acceptability Data will be examined descriptively. Client Satisfaction Questionnaire score, rates of recruitment/retention, session attendance, and completion of between session goals will be compared to relevant previous trials. Clinical outcome analyses will focus on determining if BA-HD had clinically meaningful effects. For example, we will examine within participant effect size and response rates (using standard cut offs) on the PHQ-9. We will examine within participant change in our composite of behavioral risk factors in a similar manner. We will analyze data in an intent-to-treat manner.

# d Design Considerations

Choice not to use cardiac rehabilitation. Cardiac rehabilitation is the gold standard for targeting behavioral risk factors post-ACS and also improves depression symptoms. However, only a minority of ACS patients complete cardiac rehabilitation and those with depression are particularly unlikely to attend or complete.^123^ Thus, if we conducted our treatment in a rehabilitation setting we would miss many of those with depression post-ACS. Choice to enroll participants 2-12 months after ACS. We considered enrollment of patients while in the hospital for ACS. We chose not to because: 1) there is a high rate of spontaneous recovery of hospital assessed depression in the first month post-ACS,^124^ 2) there is a high rate of development of new depression symptoms in the initial months following ACS,[^9^](#_bookmark8)[^,51^](#_bookmark23) 3) depression assessed 3 months post-ACS has specifically been linked to poor adherence to behavioral risk factors,^14^ and 4) many patients will not be cleared to increase physical activity immediately post-ACS. Choice to complete an open trial rather than an RCT. We considered conducting an RCT as part of the proposed R03. However, our primary purpose is to get as much patient feedback on the BA-HD manual as possible, making an open trail a better fit. Choice of a sequential intervention. We considered targeting depression and all relevant behavioral risk factors simultaneously.

However, simultaneous targeting of multiple risk factors is cognitively demanding for the patient and associated with increased drop-out.^125^ Given that those with depression have concentration and memory deficits, and are already more likely to drop-out of risk factor reduction treatments, we chose to target risk factors sequentially. Choice to provide free behavior change aids. Those with depression struggle with motivation, concentration, memory, and self-regulation, thus we provide reminder and goal tracking aids (e.g., wearable accelerometers, medication reminder alarms, diet measurement/tracking tools) to help address these deficits. We provide nicotine replacement treatment because smokers with depression symptoms experience more nicotine withdrawal post-ACS.^112^

# PROTECTION OF HUMAN SUBJECTS

1. **Risks to Human Subjects**

Note: The design of Aim II will be adjusted based on the results of Aim I. Thus, the protections below will be reviewed and updated as needed following the design of Aim II.

# 1a. Human Subjects Involvement, Characteristics, and Design

**AIM II** Inclusion criteria: 1) ACS diagnosis (diagnosis of unstable angina, ST and non-ST elevation myocardial infarction) documented in medical record in the preceding 2-12 months, 2) Depression diagnosis listed on patient problem list in patient’s medical record, a clinically administered PHQ-9 score of 10 or greater in the past 12 months indicated in the patient’s medical record, or a Center for Epidemiologic Studies Depression scale (CESD) score ≥10 (indicating “likely major depressive disorder”); CESD will be administered by phone during screening if there is no indication of depression or PHQ-9 score within 12 months in the patient medical record, 3) current non-adherence to ≥ 1 of four behavioral risk factors (see below), 4) willing to make immediate changes to ≥ 1 relevant behavioral risk factors, 5) age of 18-75, 6) lives within 1.5 hours of Hennepin Healthcare, and 7) fluent in English.. Exclusion criteria: 1) Limited mental competency (as indicated in medical chart), 2) presence of current exacerbation of psychosis/serious mental illness or suicidality, 3) in hospice care, 4) currently attending regular counseling targeting depression or any health behavior change, and 5) currently attending a cardiac rehabilitation program (those excluded for being in rehabilitation will be re- contacted after completion). We will attempt to obtain a sample of at least 45% women, which is representative of the gender distribution of discharged ACS patients.^1^

We will define non-adherence as follows: Tobacco non-adherence = use of any tobacco product in the last week. Physical activity non-adherence = engagement in < 90 minutes of moderate or greater physical activity per week.^104^ Medication non-adherence = non-adherence based on Heart and Soul study criteria i.e., taking medication as prescribed 75% of the time or less, forgetting medications at least 1x/week, or skipping a medication at least 1x/week. Diet non-adherence = following a “healthful eating plan” 4 or fewer days per week, eating 5 or more servings of fruits and vegetables 4 or fewer days per week, or eating high fat foods 3 or more days per week.

We are recruiting ACS patients who are depressed and struggle with at least one behavioral risk factor post-ACS because this population will be the target of our intervention. We require patient willingness to change at least one risk factor because the treatment is designed for patients with some motivation to change. We limited enrollment to ages 18-75 because young children and geriatric patients may need tailored interventions. Fluency in English is required because (at this time) the BA-HD manual and forms are only in English and clinical supervisors are mono-lingual English speakers. Those with limited mental competency, a severe mental illness that would interfere with participation, or suicidality are excluded because these groups likely require different counseling strategies and/or a higher level of counseling care than provided by the proposed intervention. Our treatment manual was not designed for use in these populations. Those in hospice are excluded because the BA-HD manual does not address end of life issues. Those currently attending counseling for depression or health behavior change are excluded because these concurrent treatments would confound primary outcomes.

For **both Aims I and II**, we will enroll individuals of all racial and ethnic backgrounds. We plan to enroll ratios of minority groups that are consistent with the population of Minneapolis, MN. According to latest United States Census data, the racial composition of individuals living in Minneapolis, MN is 63.8% White, 18.6% Black or African American, 5.6% Asian, 2.0% American Indian/Alaska Native, 0.0% Native Hawaiian/Other Pacific Islander, and 4.4% two or more races. The ethnic composition of individuals living in Minneapolis MN is 10.5% Hispanic/Latino and 86.0% Non-Hispanic/Latino. Thus, we expect that 36.2% of the sample will be members of a racial minority group, and 10.5% of the sample will be of Hispanic/Latino ethnicity. We expect to enroll a sample that is 45% women which is representative of the gender distribution of ACS patients[.^1^](#_bookmark0)[^,45^](#_bookmark22) Please see the Targeted/Planned Enrollment Table for a detailed distribution.

Enrollment, Aim II: Participants will again be recruited through review of electronic medical records at Hennepin Healthcare. We will pull records of recent ACS hospital discharges that also have depression listed as a diagnosis. The Analytics Center of Excellence (ACE) team may identify names from EPIC to facilitate this. We will also review records of outpatient cardiology and ask outpatient cardiology providers to direct us to records of patients who might qualify. A research assistant will call patients to screen for eligibility using the above criteria. In addition, patients will be invited to call a research assistant themselves via use of a recruitment flyer will be provided to cardiology patients and providers (See attached). This flyer will be given to providers for distribution to interested patients and posted in clinic waiting rooms.

Those who qualify by phone screen will be invited to an in person meeting to provide informed consent and then complete a baseline assessment. The research assistant will then schedule the patient for a first session of BA-HD coaching at the Berman center or other Hennepin Healthcare facility (or in patient’s home when needed).

Procedure, Aim II: Following informed consent, participants will complete self-report, interview, and anthropomorphic measures at enrollment and at end-of-treatment (i.e., following 12 weeks of BA-HD coaching). In addition, at end-of-treatment a 30 minute qualitative interview will be conducted. Qualitative questions will focus on the participants experience throughout the study (e.g., How was the screening process? How was the consent and enrollment process? What did you like and dislike about the coaching program?, etc). Data will also be pulled from the medical chart. Participants will be compensated $30 per assessment and an extra $20 for completion of the qualitative interview. Thus, participants completing could receive $80 total for assessment completion. Transportation will also be provided or reimbursed as well.

Consistent with successful BA manuals and our Aim 1 qualitative data, we plan to conduct a 10 session treatment over 12 weeks (weekly for the first 8 sessions and bi-weekly for the final two). The initial two sessions will be about 50 minutes long and later sessions will be 20-30 minutes long. We plan a treatment that is primarily over the phone, but allowing for the 1^st^ and 2^nd^ sessions in person and in home if needed per patient preferences. Dr. Busch, a Hennepin Healthcare employed exercise physiologist, and/or an HHRI employed social worker will conduct all treatment in the proposed study.

# 1b. Sources of Materials

All data collection will follow IRB and HIPAA guidelines. Data will be collected directly from participants and from medical records. Data will include participant responses to interviews and self-report questionnaires (including screening questions), expired air samples (aim II for those focused on tobacco cessation only, i.e., carbon monoxide testing), physical measurements (aim II only, e.g., blood pressure, height, weight), and data pulled from medical charts. Coaching sessions (aim II only) and qualitative interviews (aim I only) will also be audio recorded (explicit permission to do so will be obtained).

As participants will be recruited via chart review (medical records will be screened in order to identify patients who may meet inclusion criteria), we will apply for a Protected Health Information (PHI) waiver from our IRB. All data will be treated as confidential and will never be stored or reported in association with identifying information. Participant responses to interviews and self-report questionnaires and information obtained from medical charts will be entered directly into a secure electronic database. Only the PI, Co-Is, and study staff that have completed training in the ethical conduct of research will obtain or have access to identifiable information. All data will be de-identified and filed according to participant ID numbers in secure storage. Any forms with identifying information (e.g., signed informed consent forms) will be stored separately. Only de-identified data will be published.

# 1c. Potential Risks

Nicotine replacement side effects (Aim II only): All subjects in Aim II who are tobacco users, ready to quit in 30 days, and cleared for use by the study team will be offered a free course of nicotine patches and/or the nicotine lozenge. Nicotine replacement treatment has been in clinical use for 20 years. It is available over the counter and has been judged to be generally safe and efficacious with very few contraindications. Nicotine replacement has been found to be medically safe for use with cardiac patients,^126^ including specifically among ACS patients[.^127^](#_bookmark48)[^,128^](#_bookmark49) Nicotine replacement is medically indicated for the vast majority of smokers with few exceptions (e.g., pregnancy). However, there are some reports indicating that nicotine replacement use poses risk to some subgroups of ACS patients in some situations^129^. Thus, if nicotine replacement is provided, the dose will be determined in consultation with study team (i.e., Dr. Busch: psychologist/tobacco expert; Dr.

Ayenew: cardiologist; Dr. Vickery: family physician). The patient’s primary provider will be informed through EPIC messaging that nicotine replacement was provided to the participant. The study MDs/PhD may talk to the patient to obtain more information in order to assess appropriateness of nicotine replacement. Initial dosing will follow FDA instructions/clinical guidelines, but lower doses may be provided if recommended by Drs. Busch, Ayenew, or Vickery. Staff will be trained to explain the purpose of nicotine replacement, use, and side effects. Common side effects of the patch include local skin irritation at the site of the patch and disturbed or vivid dreams. Common side effects of the nicotine lozenge include mouth problems, indigestion, and sore throat.

Less common reactions to both the patch and lozenge includes allergic reactions. If the dose of nicotine replacement is too large or if the participant continues to smoke at a high level while using the nicotine replacement, they may experience symptoms of nicotine overuse including nausea, irregular heartbeat or palpitation, and dizziness. The risk of using nicotine replacement in the current study is judged to be very minor.

Nicotine Withdrawal Symptoms After Quitting (Aim II only): There is a strong likelihood that most study participants who quit smoking will experience some nicotine withdrawal symptoms, including anxiety, restlessness, anger, irritability, sadness, problems concentrating, appetite change and weight gain, insomnia, and decreased heart rate. Generally, these reactions are temporary and pose no serious health risks.

Injury from increasing physical activity (Aim II only): It is possible that physical activity post-ACS could trigger a recurrent cardiac event or cause other injury. The risk of injury from increasing physical activity in the current study is judged to be very minor, given that we will focus on increasing walking (rather than more intense exercise).

Confidentiality or loss of privacy. We will collect potentially sensitive information about participants; if released inappropriately, participants may experience embarrassment or distress. The seriousness of the consequences would depend on the nature of the information revealed and to whom the information was revealed. Given the numerous steps we take to protect participant confidentiality, we think the risk of a breach of confidentiality is low.

Discomfort or distress when completing assessment and treatment procedures. Some participants may feel uncomfortable or distressed answering personal or private questions during assessment or treatment.

Some participants may also feel uncomfortable or distressed due to the collection of physical measures (e.g., weight). In our previous studies, when individuals did report discomfort in these situations, it was mild.

Worsening of depression and emergent suicidality. The post-ACS period can be a high stress time. Although not likely resulting from trial participation, it is likely that a minority of participants will experience worsening of depression or episodes of suicidality during this study.

# Adequacy of Protection Against Risk 2a. Recruitment and Informed Consent

Participants who appear to meet inclusion/exclusion criteria, based on review of their electronic medical

records, will be called by study staff. At first contact, all participants will first be given a brief verbal overview of the study. If participants agree to be screened and pass the screening questions based on inclusion/exclusion criteria they will be invited to participate. Written informed consent (including a description of the nature, purpose, risks, and benefits of the study) will be obtained from participants before initiating assessment. The voluntary nature of the study and the participant’s right to withdrawal at any time will be stressed during the consent process; this information will be provided to participants in written form at the time of consent. As part of the consent process for Aim II, we will also ask five questions about the study. The staff member will clarify any misunderstandings that emerge during this process. Those who answer fewer than four questions correctly (after two tries) will be considered to lack the capacity to consent to the research. Research assistants and study interventionists will obtain consent.

# 2b. Protections Against Risk

Minimization of nicotine replacement side effects: Nicotine replacement is generally safe for patients with stable cardiac disease^126^ and with post-ACS patients[.^132^](#_bookmark51)[^,133^](#_bookmark52) However, there are some reports indicating that nicotine replacement use poses risk to some subgroups of ACS patients in some situations^129^. **Thus, we will minimize the occurrence of any serious medical side effects by having the** study provider team (i.e., Dr. Busch: psychologist/tobacco expert; Dr. Ayenew: cardiologist; Dr. Vickery: family physician) review if the patient is appropriate for nicotine replacement and if so what the dose should be. Initial dosing will follow FDA instructions and clinical guidelines, but lower doses may be provided if recommended by the study team.

Participants will agree to consult with their outpatient medical providers regarding the safety of using nicotine replacement if their medical status changes after nicotine replacement is initially provided. Further, staff will be trained to explain the purpose of nicotine replacement, procedure for use, and side effects, as well as how to use nicotine replacement in a manner that minimizes risk.

To minimize skin reactions related to the nicotine patch, participants will be instructed to move the site of patch placement each day and to not repeat site use for at least one week. Smokers will be instructed to remove the patch before bed if it significantly interferes with sleep. To minimize lozenge side effects, participants will be instructed in proper use in detail (i.e., do not chew the lozenge).

Nicotine replacement dose may be adjusted downward if there is significant nausea or other reactions.

Participants with severe side effects will be asked to discontinue nicotine replacement use and discuss with study team and/or their providers before continuing.

Only participants who are ready to quit within 30 days will be provided with nicotine replacement.

Participants will be allowed to use the nicotine lozenge to reduce smoking in preparation for a quit day (i.e., consistent with a “cut down to quit” approach.

Minimization of nicotine withdrawal symptoms after quitting: Participants who decide to use the nicotine replacement will be told that it will reduce but not entirely eliminate withdrawal symptoms. Participants will be instructed to call their outpatient physician in the case of severe withdrawal reactions. Withdrawal symptoms typically abate within 1 to 2 weeks of quitting and are not medically dangerous.

Minimization of potential for injury from increasing physical activity. BA-HD focuses on increasing self- paced walking (i.e., goals well be stated in steps per day on wearable accelerometers/pedometers), which is safe for the vast majority of patients who are ≥3 months post-ACS. If the interventionist has any concern regarding the safety of walking Dr. Ayenew and Dr. Vickery will be available for consultation. The patient may want to work on more intense exercise, especially later in the treatment protocol. If this is the case the interventionist will discuss with the patient if they have been cleared by their medical team to engage in the desired activity. If there is any concern regarding clearance for exercise, the interventionist may contact the patient’s providers and/or consult with Dr. Ayenew and Dr. Vickery.

Minimization of loss of confidentiality/privacy: All data and records will be safeguarded according to the strict privacy/confidentiality policies of Hennepin Healthcare Research Institute’s Institutional Review Board (IRB). Confidentiality will be maintained by numerically coding all data, disguising identifying information, and keeping data in secure electronic locations or locked in file drawers. All electronic data will be numerically coded and stored on a password protected computer in a secure research space. All paper forms will be stored in locked file cabinets in a locked room. Names of participants will be stored separately. Participant information will be accessible only to research staff, who are pledged to confidentiality and complete training in the ethical

conduct of research (i.e., both HIPAA and CITI trainings). Dr. Busch will also personally train staff on maintenance of participant confidentiality. Identifying information will not be reported in any publication.

Minimization of discomfort or distress when completing assessment and treatment procedures: We will take three specific steps to reduce the possibility of discomfort or distress:

*Study will be clearly explained*. A detailed explanation of the study, including what study participation would involve, the nature of the questions participants will be asked to answer, the nature of measurements, and the right to withdrawal from the study at any time without penalty, will be provided to the participants, both verbally and in writing (through the informed consent form). Participants will be encouraged to ask questions about the study. Individuals who are uncomfortable answering these types of questions, assessments, or interventions likely will not choose to participate. Those who choose to participate but are very uncomfortable with the questions, assessments or interventions will likely refuse these assessments or choose to withdrawal from the study.

*Private setting*. To help ensure clients’ comfort, they will answer survey questions and complete physical measurements in a private setting. Thus, they should not be concerned that another individual would be able to observe these assessments.

*Staff Training***.** All staff interacting with participants will be trained by Dr. Busch to ask questions and complete assessments in a sensitive manner and be supportive to any participant experiencing discomfort or distress.

Minimization of risk from worsening of depression and emergent suicidality. **There is no evidence indicating that participation in this trial will worsen depression or cause suicidality.** However, given that post-ACS period can be a high stress time, it is likely that a minority of participants will experience worsening of depression during this study. A smaller minority may experience episodes of suicidality. Thus, we will monitor and respond to these issues in an ethically sensitive manner.

For aim II, we are only enrolling participants who are not currently in counseling for depression.

Participants taking an anti-depressant medication at baseline and during the study will be allowed to participate. For ethical reasons, once enrolled we will not restrict anti-depressant medication treatment seeking in any way during the study (i.e., we will not ask participants to refrain from starting antidepressant medication during the study). We will carefully track antidepressant medication seeking outside of the study and will control for outside treatment in analyses as appropriate.

All participants will be assessed for depression using the PHQ-9 at all assessments. If a participant continues to report significant depression (i.e., PHQ-9 ≥10) at the end-of-treatment assessment, staff will 1) encourage the participant to discuss these symptoms with his or her medical providers and 2) provide a list of community psychiatric treatment referrals.

If a participant reports active suicidality (i.e., any recent suicidal attempts, suicidal gestures, or self- injurious behavior; any current plan or intent to engage in suicidal or self-injurious behavior) to any study staff at any time point, the participant will be assessed by Dr. Busch (a licensed clinical psychologist). Dr. Busch will assess the participant and respond in an appropriate and ethical manner according to the standards of care for suicide prevention (e.g., referral to immediate emergency psychiatric care if danger to self is imminent).

# Potential Benefits of the Proposed Research

Potential benefits for Aim II participants include free treatment with the potential to increase the likelihood of behavioral risk factor reduction, which could in turn extend their lives. Aim II participants will also receive free depression coaching, which can improve their quality of life. By participating in the research, all participants (both Aims I and II) will also benefit from knowing they may ultimately be helping others as they will have helped us develop and test an intervention. The costs of participating in the research will be minimized through our extensive efforts to maintain confidentiality, reduce discomfort or distress, and minimize medical complications. Overall, it is expected that the potential benefits to participants in the proposed study will outweigh potential risks.

# Importance of the Knowledge to be Gained

The results of the proposed study will further our knowledge regarding 1) the relationship between behavioral risk factors and mood in post-ACS patients and 2) the utility of integrating Behavioral Activation based depression treatment with behavioral risk factor reduction for cardiac patients. This knowledge could lead to a reduction in post-ACS mortality rates. As discussed above, we expect that these benefits will outweigh potential risks to participants.

# Data and Safety Monitoring Plan

We expect the proposed trial to be considered minimal risk by Hennepin Healthcare Research Institute’s IRB. Given that the proposed Aim II research is a single site trial and our expectation of a minimal risk designation, the proposed trial does not meet NIH criteria requiring establishment of a formal Data and Safety Monitoring Board. However, we have a detailed data and safety monitoring plan for aim II to ensure the safety of all participants and the validity and integrity of the data. (Note: If our Institutional Review Board or the study sponsor unexpectedly decides that the proposed study includes more than minimal risk to participants, we will convene a Data and Safety Monitoring Board before recruitment begins.) The Data and Safety Monitoring plan is provided in a separate attachment.

# ClinicalTrials.gov Requirements

The proposed clinical trial will be registered at [www.clinicaltrials.gov.](http://www.clinicaltrials.gov/)

**Amendment 2: H3 Study Issued Cell Phone Protocol**

If at time of recruitment or any time during treatment/follow-up study staff determine that the participant does not have access to or has lost access to regular phone access, the participant may be offered use of a H^3^ provided cell phone. Participants who are issued a study phone will complete the H^3^ Study Issued Cell Phone Agreement form. Patient will be instructed to return the H^3^ provided phone to staff upon completion of the 12w outcomes visit or at any time if it is determined that the participant no longer qualifies for the study or is not adhering to the guidelines outlined in the H^3^ Study Issued Cell Phone Agreement. Upon return of the device, participants will qualify for a $20 stipend.

*See Appendix A: Study Issued Cell Phone Agreement.*

**References**

1. **Go AS, Mozaffarian D, Roger VL, et al. Heart disease and stroke statistics--2014 update: a report from the American Heart Association. *Circulation.* Jan 21 2014;129(3):e28-e292.**
2. **Lichtenstein AH, Appel LJ, Brands M, et al. Diet and lifestyle recommendations revision 2006: a scientific statement from the American Heart Association Nutrition Committee. *Circulation.* Jul 4** **2006;114(1):82-96.**
3. **Jensen MK, Chiuve SE, Rimm EB, et al. Obesity, behavioral lifestyle factors, and risk of acute coronary events. *Circulation.* Jun 17 2008;117(24):3062-3069.**
4. **Mohiuddin SM, Mooss AN, Hunter CB, Grollmes TL, Cloutier DA, Hilleman DE. Intensive smoking cessation intervention reduces mortality in high-risk smokers with cardiovascular** **disease. *Chest.* Feb 2007;131(2):446-452.**
5. **Eisenberg MJ, Grandi SM, Gervais A, et al. Bupropion for smoking cessation in patients hospitalized with acute myocardial infarction: a randomized, placebo-controlled trial. *Journal of the American College of Cardiology.* Feb 5 2013;61(5):524-532.**
6. **Desai NR, Choudhry NK. Impediments to adherence to post myocardial infarction medications.**

***Curr Cardiol Rep.* Jan 2013;15(1):322.**

1. **Cheng K, Ingram N, Keenan J, Choudhury RP. Evidence of poor adherence to secondary prevention after acute coronary syndromes: possible remedies through the application of new technologies. *Open Heart.* 2015;2(1):e000166.**
2. **Chow CK, Jolly S, Rao-Melacini P, Fox KA, Anand SS, Yusuf S. Association of diet, exercise, and smoking modification with risk of early cardiovascular events after acute coronary syndromes. *Circulation.* Feb 16 2010;121(6):750-758.**
3. **Thombs BD, Bass EB, Ford DE, et al. Prevalence of depression in survivors of acute myocardial infarction. *J Gen Intern Med.* Jan 2006;21(1):30-38.**
4. **Lichtman JH, Froelicher ES, Blumenthal JA, et al. Depression as a risk factor for poor prognosis among patients with acute coronary syndrome: systematic review and recommendations: a scientific statement from the American Heart Association. *Circulation.* Mar 25 2014;129(12):1350- 1369.**
5. **Meijer A, Conradi HJ, Bos EH, Thombs BD, van Melle JP, de Jonge P. Prognostic association of depression following myocardial infarction with mortality and cardiovascular events: a meta-** **analysis of 25 years of research. *General hospital psychiatry.* May-Jun 2011;33(3):203-216.**
6. **van Melle JP, de Jonge P, Spijkerman TA, et al. Prognostic association of depression following myocardial infarction with mortality and cardiovascular events: a meta-analysis. *Psychosom Med.* Nov-Dec 2004;66(6):814-822.**
7. **Doyle F, Rohde D, Rutkowska A, Morgan K, Cousins G, McGee H. Systematic review and meta- analysis of the impact of depression on subsequent smoking cessation in patients with coronary heart disease: 1990 to 2013. *Psychosom Med.* Jan 2014;76(1):44-57.**
8. **Kronish IM, Rieckmann N, Halm EA, et al. Persistent depression affects adherence to secondary prevention behaviors after acute coronary syndromes. *J Gen Intern Med.* Nov 2006;21(11):1178-** **1183.**
9. **Ye S, Muntner P, Shimbo D, et al. Behavioral mechanisms, elevated depressive symptoms, and the risk for myocardial infarction or death in individuals with coronary heart disease: the REGARDS (Reason for Geographic and Racial Differences in Stroke) study. *Journal of the American College of Cardiology.* Feb 12 2013;61(6):622-630.**
10. **Chrysohoou C, Liontou C, Aggelopoulos P, et al. Mediterranean diet mediates the adverse effect of depressive symptomatology on short-term outcome in elderly survivors from an acute coronary event. *Cardiol Res Pract.* 2011;2011:429487.**
11. **Brummett BH, Babyak MA, Siegler IC, Mark DB, Williams RB, Barefoot JC. Effect of smoking and sedentary behavior on the association between depressive symptoms and mortality from coronary heart disease. *The American journal of cardiology.* Sep 1 2003;92(5):529-532.**
12. **Kronish IM, Rieckmann N, Burg MM, Edmondson D, Schwartz JE, Davidson KW. The effect of enhanced depression care on adherence to risk-reducing behaviors after acute coronary syndromes: findings from the COPES trial. *Am Heart J.* Oct 2012;164(4):524-529.**
13. **Berkman LF, Blumenthal J, Burg M, et al. Effects of treating depression and low perceived social support on clinical events after myocardial infarction: the Enhancing Recovery in Coronary Heart Disease Patients (ENRICHD) Randomized Trial. *JAMA.* Jun 18 2003;289(23):3106-3116.**
14. **O'Neil A, Hawkes AL, Atherton JJ, et al. Telephone-delivered health coaching improves anxiety outcomes after myocardial infarction: the 'ProActive Heart' trial. *Eur J Prev Cardiol.* Jan** **2014;21(1):30-38.**
15. **O'Neil A, Taylor B, Hare DL, et al. Long-term efficacy of a tele-health intervention for acute coronary syndrome patients with depression: 12-month results of the MoodCare randomized controlled trial. *Eur J Prev Cardiol.* Sep 2015;22(9):1111-1120.**
16. **Mitchell PH, Veith RC, Becker KJ, et al. Brief psychosocial-behavioral intervention with antidepressant reduces poststroke depression significantly more than usual care with antidepressant: living well with stroke: randomized, controlled trial. *Stroke; a journal of cerebral circulation.* Sep 2009;40(9):3073-3078.**
17. **Pagoto S, Schneider KL, Whited MC, et al. Randomized controlled trial of behavioral treatment for comorbid obesity and depression in women: the Be Active Trial. *Int J Obes (Lond).* Nov 2013;37(11):1427-1434.**
18. **Hopko DR, Armento ME, Robertson SM, et al. Brief behavioral activation and problem-solving therapy for depressed breast cancer patients: randomized trial. *Journal of consulting and clinical psychology.* Dec 2011;79(6):834-849.**
19. **Dimidjian S, Barrera M, Jr., Martell C, Munoz RF, Lewinsohn PM. The origins and current** **status of behavioral activation treatments for depression. *Annu Rev Clin Psychol.* 2011;7:1-38.**
20. **Dimidjian S, Hollon SD, Dobson KS, et al. Randomized trial of behavioral activation, cognitive therapy, and antidepressant medication in the acute treatment of adults with major depression. *J Consult Clin Psychol.* Aug 2006;74(4):658-670.**
21. **Ekers D, Richards D, Gilbody S. A meta-analysis of randomized trials of behavioural treatment of depression. *Psychological medicine.* May 2008;38(5):611-623.**
22. **Ekers D, Webster L, Van Straten A, Cuijpers P, Richards D, Gilbody S. Behavioural activation for depression; an update of meta-analysis of effectiveness and sub group analysis. *PloS one.* 2014;9(6):e100100.**
23. **Alexopoulos GS, Raue PJ, Kiosses DN, Seirup JK, Banerjee S, Arean PA. Comparing engage with PST in late-life major depression: a preliminary report. *Am J Geriatr Psychiatry.* May 2015;23(5):506-513.**
24. **Ekers D, Dawson MS, Bailey E. Dissemination of behavioural activation for depression to mental health nurses: training evaluation and benchmarked clinical outcomes. *J Psychiatr Ment Health Nurs.* Mar 2013;20(2):186-192.**
25. **Ekers D, Richards D, McMillan D, Bland JM, Gilbody S. Behavioural activation delivered by the nonspecialist: Phase II randomised controlled trial. *The British Journal of Psychiatry.* 2011;198(1):66-72.**
26. **Hollon SD. Behavioral activation treatment for depression: A commentary. *Clinical Psychology:*** ***Science and Practice.* 2001;8:271-274.**
27. **Chowdhary N, Anand A, Dimidjian S, et al. The Healthy Activity Program lay counsellor delivered treatment for severe depression in India: systematic development and randomised evaluation. *Br J Psychiatry.* Apr 2016;208(4):381-388.**
28. **Daughters SB, Braun AR, Sargeant MN, et al. Effectiveness of a brief behavioral treatment for inner-city illicit drug users with elevated depressive symptoms: the life enhancement treatment for substance use (LETS Act!). *The Journal of clinical psychiatry.* Jan 2008;69(1):122-129.**
29. **Daughters SB, Magidson JF, Schuster RM, Safren SA. ACT HEALTHY: A Combined Cognitive- Behavioral Depression and Medication Adherence Treatment for HIV-Infected Substance Users. *Cogn Behav Pract.* Aug 1 2010;17(3):309-321.**
30. **Mimiaga MJ, Reisner SL, Pantalone DW, O'Cleirigh C, Mayer KH, Safren SA. A pilot trial of integrated behavioral activation and sexual risk reduction counseling for HIV-uninfected men who have sex with men abusing crystal methamphetamine. *AIDS Patient Care STDS.* Nov 2012;26(11):681-693.**
31. **Magidson JF, Gorka SM, MacPherson L, et al. Examining the effect of the Life Enhancement Treatment for Substance Use (LETS ACT) on residential substance abuse treatment retention. *Addictive behaviors.* Jun 2011;36(6):615-623.**
32. **Pagoto SL, Schneider K, Olendzki B, Spates CR, Ma Y. Initial investigation of behavioral activation treatment for comorbid major depressive disorder and obesity. *Psychotherapy: Theory, Research, Practice, Training.* 2008;45(3):410-415.**
33. **MacPherson L, Tull MT, Matusiewicz AK, et al. Randomized controlled trial of behavioral activation smoking cessation treatment for smokers with elevated depressive symptoms. *Journal of consulting and clinical psychology.* Feb 2010;78(1):55-61.**
34. **Banducci AN, Lejuez CW, MacPherson L. Pilot of a Behavioral Activation-Enhanced Smoking Cessation Program for Substance Users With Elevated Depressive Symptoms in Residential Treatment. *Addictions newsletter.* Summer 2013;2013:16-20.**
35. **Davidson KW, Rieckmann N, Clemow L, et al. Enhanced depression care for patients with acute coronary syndrome and persistent depressive symptoms: coronary psychosocial evaluation studies randomized controlled trial. *Arch Intern Med.* Apr 12 2010;170(7):600-608.**
36. **Davidson KW, Bigger JT, Burg MM, et al. Centralized, stepped, patient preference-based treatment for patients with post-acute coronary syndrome depression: CODIACS vanguard randomized controlled trial. *JAMA internal medicine.* Jun 10 2013;173(11):997-1004.**
37. **Huffman JC, Mastromauro CA, Sowden GL, Wittmann C, Rodman R, Januzzi JL. A collaborative care depression management program for cardiac inpatients: depression characteristics and in-hospital outcomes. *Psychosomatics.* Jan-Feb 2011;52(1):26-33.**
38. **Huffman JC, Mastromauro CA, Sowden G, Fricchione GL, Healy BC, Januzzi JL. Impact of a depression care management program for hospitalized cardiac patients. *Circulation. Cardiovascular quality and outcomes.* Mar 2011;4(2):198-205.**
39. **Khot UN, Khot MB, Bajzer CT, et al. Prevalence of conventional risk factors in patients with coronary heart disease. *JAMA.* Aug 20 2003;290(7):898-904.**
40. **Control CfD. Cigarette Smoking Among Adults- United States, 2006. *Morbidity and Mortality Weekly Report.* 2007;56:1157-1161.**
41. **Shahoumian TA, Phillips BR, Backus LI. Cigarette Smoking, Reduction and Quit Attempts: Prevalence Among Veterans With Coronary Heart Disease. *Prev Chronic Dis.* 2016;13:E41.**
42. **Rigotti NA, Munafo MR, Stead LF. Smoking cessation interventions for hospitalized smokers: a systematic review. *Archives of internal medicine.* Oct 13 2008;168(18):1950-1960.**
43. **Dawood N, Vaccarino V, Reid KJ, Spertus JA, Hamid N, Parashar S. Predictors of smoking cessation after a myocardial infarction: the role of institutional smoking cessation programs in improving success. *Arch Intern Med.* Oct 13 2008;168(18):1961-1967.**
44. **Prugger C, Wellmann J, Heidrich J, et al. Readiness for smoking cessation in coronary heart disease patients across Europe: Results from the EUROASPIRE III survey. *Eur J Prev Cardiol.* Dec 16 2014.**
45. **Parker GB, Hilton TM, Walsh WF, et al. Timing is everything: the onset of depression and acute** **coronary syndrome outcome. *Biological psychiatry.* Oct 15 2008;64(8):660-666.**
46. **Zuidersma M, Thombs BD, de Jonge P. Onset and recurrence of depression as predictors of cardiovascular prognosis in depressed acute coronary syndrome patients: a systematic review. *Psychother Psychosom.* 2011;80(4):227-237.**
47. **Anda RF, Williamson DF, Escobedo LG, Mast EE, Giovino GA, Remington PL. Depression and** **the dynamics of smoking. A national perspective. *JAMA.* Sep 26 1990;264(12):1541-1545.**
48. **Wilhelm K, Mitchell P, Slade T, Brownhill S, Andrews G. Prevalence and correlates of DSM-IV major depression in an Australian national survey. *Journal of affective disorders.* Jul 2003;75(2):155-162.**
49. **Sieverdes JC, Ray BM, Sui X, et al. Association between leisure time physical activity and** **depressive symptoms in men. *Medicine and science in sports and exercise.* Feb 2012;44(2):260-265.**
50. **Farmer M, Locke B, Moscicki E, Dannenberg A, Larson D, Radloff L. Physical activity and depressive symptoms: the NHANES I Epidemiologic Follow-up Study. *American Journal of Epidemiology.* 1988;128(6):1340-1351.**
51. **Akbaraly TN, Brunner EJ, Ferrie JE, Marmot MG, Kivimaki M, Singh-Manoux A. Dietary pattern and depressive symptoms in middle age. *Br J Psychiatry.* Nov 2009;195(5):408-413.**
52. **Luppino FS, de Wit LM, Bouvy PF, et al. Overweight, obesity, and depression: a systematic review and meta-analysis of longitudinal studies. *Arch Gen Psychiatry.* Mar 2010;67(3):220-229.**
53. **Grenard JL, Munjas BA, Adams JL, et al. Depression and medication adherence in the treatment of chronic diseases in the United States: a meta-analysis. *Journal of general internal medicine.* Oct** **2011;26(10):1175-1182.**
54. **DiMatteo MR, Lepper HS, Croghan TW. Depression is a risk factor for noncompliance with medical treatment: meta-analysis of the effects of anxiety and depression on patient adherence. *Archives of internal medicine.* Jul 24 2000;160(14):2101-2107.**
55. **Kahler CW, Brown RA, Ramsey SE, et al. Negative mood, depressive symptoms, and major depression after smoking cessation treatment in smokers with a history of major depressive disorder. *Journal of abnormal psychology.* Nov 2002;111(4):670-675.**
56. **Niaura R, Britt DM, Shadel WG, Goldstein M, Abrams D, Brown R. Symptoms of depression and survival experience among three samples of smokers trying to quit. *Psychol Addict Behav.* Mar 2001;15(1):13-17.**
57. **Gonzalez JS, Peyrot M, McCarl LA, et al. Depression and diabetes treatment nonadherence: a meta-analysis. *Diabetes care.* Dec 2008;31(12):2398-2403.**
58. **Wing RR, Phelan S, Tate D. The role of adherence in mediating the relationship between depression and health outcomes. *J Psychosom Res.* Oct 2002;53(4):877-881.**
59. **Mazzeschi C, Pazzagli C, Buratta L, et al. Mutual interactions between depression/quality of life and adherence to a multidisciplinary lifestyle intervention in obesity. *The Journal of clinical endocrinology and metabolism.* Dec 2012;97(12):E2261-2265.**
60. **Krogh J, Videbech P, Thomsen C, Gluud C, Nordentoft M. DEMO-II trial. Aerobic exercise versus stretching exercise in patients with major depression-a randomised clinical trial. *PloS one.* 2012;7(10):e48316.**
61. **Busch AM, Scott-Sheldon LA, Pierce J, et al. Depressed mood predicts pulmonary rehabilitation completion among women, but not men. *Respir Med.* Jul 2014;108(7):1007-1013.**
62. **Ellard DR, Thorogood M, Underwood M, Seale C, Taylor SJ. Whole home exercise intervention for depression in older care home residents (the OPERA study): a process evaluation. *BMC*** ***medicine.* 2014;12:1.**
63. **Myers V, Gerber Y, Benyamini Y, Goldbourt U, Drory Y. Post-myocardial infarction depression: increased hospital admissions and reduced adoption of secondary prevention measures--a longitudinal study. *J Psychosom Res.* Jan 2012;72(1):5-10.**
64. **Ziegelstein RC, Fauerbach JA, Stevens SS, Romanelli J, Richter DP, Bush DE. Patients with depression are less likely to follow recommendations to reduce cardiac risk during recovery from a myocardial infarction. *Arch Intern Med.* Jun 26 2000;160(12):1818-1823.**
65. **McGee HM, Doyle F, Conroy RM, De La Harpe D, Shelley E. Impact of briefly-assessed depression on secondary prevention outcomes after acute coronary syndrome: a one-year longitudinal survey. *BMC Health Serv Res.* 2006;6:9.**
66. **Whooley MA, de Jonge P, Vittinghoff E, et al. Depressive symptoms, health behaviors, and risk of cardiovascular events in patients with coronary heart disease. *JAMA.* Nov 26 2008;300(20):2379- 2388.**
67. **O'Neil A, Taylor B, Sanderson K, et al. Efficacy and feasibility of a tele-health intervention for acute coronary syndrome patients with depression: results of the "MoodCare" randomized controlled trial. *Annals of behavioral medicine : a publication of the Society of Behavioral Medicine.* Oct 2014;48(2):163-174.**
68. **Hollon SD. Do cognitive change strategies matter in cognitive therapy? . *Prevention & Treatment.***

**2000;3(Article 25).**

1. **Kanter JW, Busch, A. M. & Rusch, L. C. *Behavioral Activation: Distinctive Features*. London:** **Routledge Press; 2009.**
2. **Sturmey P. Behavioral activation is an evidence-based treatment for depression. *Behavior modification.* Nov 2009;33(6):818-829.**
3. **Moore RC, Chattillion EA, Ceglowski J, et al. A randomized clinical trial of Behavioral Activation (BA) therapy for improving psychological and physical health in dementia caregivers: results of** **the Pleasant Events Program (PEP). *Behaviour research and therapy.* Oct 2013;51(10):623-632.**
4. **Ekers DM, Dawson MS, Bailey E. Dissemination of behavioural activation for depression to mental health nurses: training evaluation and benchmarked clinical outcomes. *J Psychiatr Ment Health Nurs.* Mar 2013;20(2):186-192.**
5. **Rovner BW, Casten RJ, Hegel MT, et al. Low vision depression prevention trial in age-related macular degeneration: a randomized clinical trial. *Ophthalmology.* Nov 2014;121(11):2204-2211.**
6. **Doyle F, McGee HM, Conroy RM, Delaney M. What predicts depression in cardiac patients: sociodemographic factors, disease severity or theoretical vulnerabilities? *Psychol Health.* May 2011;26(5):619-634.**
7. **Rieckmann N, Burg MM, Gerin W, Chaplin WF, Clemow L, Davidson KW. Depression vulnerabilities in patients with different levels of depressive symptoms after acute coronary syndromes. *Psychotherapy and psychosomatics.* 2006;75(6):353-361.**
8. **Denton EG, Rieckmann N, Davidson KW, Chaplin WF. Psychosocial vulnerabilities to depression after acute coronary syndrome: the pivotal role of rumination in predicting and maintaining depression. *Front Psychol.* 2012;3:288.**
9. **Doyle F, McGee H, Delaney M, Motterlini N, Conroy R. Depressive vulnerabilities predict depression status and trajectories of depression over 1 year in persons with acute coronary syndrome. *General hospital psychiatry.* May-Jun 2011;33(3):224-231.**
10. **von Kanel R, Mausbach BT, Mills PJ, et al. Longitudinal relationship of low leisure satisfaction but not depressive symptoms with systemic low-grade inflammation in dementia caregivers. *J Gerontol B Psychol Sci Soc Sci.* May 2014;69(3):397-407.**
11. **Mausbach BT, Chattillion E, Roepke SK, et al. A longitudinal analysis of the relations among stress, depressive symptoms, leisure satisfaction, and endothelial function in caregivers. *Health psychology : official journal of the Division of Health Psychology, American Psychological Association.* Jul 2012;31(4):433-440.**
12. **Chattillion EA, Ceglowski J, Roepke SK, et al. Pleasant events, activity restriction, and blood pressure in dementia caregivers. *Health psychology : official journal of the Division of Health Psychology, American Psychological Association.* Jul 2013;32(7):793-801.**
13. **Busch AM, Whited MC, Appelhans BM, et al. Reliable change in depression during behavioral weight loss treatment among women with major depression. *Obesity.* 2013;21(3):E211-E218.**
14. **Busch AM, Borrelli B, Leventhal AM. The Relationship between Smoking and Depression Post- Acute Coronary Syndrome. *Curr Cardiovasc Risk Rep.* Feb 1 2012;5(6):510-518.**
15. **Busch AM, Fani Srour J, Arrighi JA, Kahler CW, Borrelli B. Valued Life Activities, Smoking Cessation, and Mood in Post-Acute Coronary Syndrome Patients. *Int J Behav Med.* Dec 4 2014.**
16. **Busch AM, Scott-Sheldon LA, Pierce J, et al. Depressed mood predicts pulmonary rehabilitation completion among women, but not men. *Respir Med.* Jul 2014;108(7):1007-1013.**
17. **Busch AM, Wagener TL, Gregor KL, Ring KT, Borrelli B. Utilizing reliable and clinically significant change criteria to assess for the development of depression during smoking cessation treatment: the importance of tracking idiographic change. *Addictive behaviors.* Dec 2011;36(12):1228-1232.**
18. **Busch AM, Whited MC, Appelhans BM, et al. Reliable change in depression during behavioral weight loss treatment among women with major depression. *Obesity (Silver Spring).* Mar 2013;21(3):E211-218.**
19. **Kahler CW, Spillane NS, Busch AM, Leventhal AM. Time-varying smoking abstinence predicts lower depressive symptoms following smoking cessation treatment. *Nicotine Tob Res.* Feb 2011;13(2):146-150.**
20. **Nguyen TD, Attkisson CC, Stegner BL. Assessment of patient satisfaction: development and refinement of a service evaluation questionnaire. *Evaluation and program planning.* 1983;6(3-** **4):299-313.**
21. **Cox DR. Regression models and life tables (with discussion). *Journal of the Royal Statistical Society Series B (Methodological).* 1972;34:187-220.**
22. **Hosmer DW, Lemeshow S. *Applied survival analysis*. Wiley Series in Probability and Statistics. Hoboken, NJ: John Wiley & Sons; 1999.**
23. **Kroenke K, Spitzer RL, Williams JB. The PHQ-9: validity of a brief depression severity measure.**

***J Gen Intern Med.* Sep 2001;16(9):606-613.**

1. **Rutledge T, Redwine LS, Linke SE, Mills PJ. A meta-analysis of mental health treatments and cardiac rehabilitation for improving clinical outcomes and depression among patients with coronary heart disease. *Psychosomatic medicine.* May 2013;75(4):335-349.**
2. **Katon WJ, Lin EH, Von Korff M, et al. Collaborative care for patients with depression and** **chronic illnesses. *The New England journal of medicine.* Dec 30 2010;363(27):2611-2620.**
3. **Redfern J, Briffa T, Ellis E, Freedman SB. Choice of secondary prevention improves risk factors after acute coronary syndrome: 1-year follow-up of the CHOICE (Choice of Health Options In prevention of Cardiovascular Events) randomised controlled trial. *Heart.* Mar 2009;95(6):468-475.**
4. **Folstein MF, Folstein SE, McHugh PR. "Mini-mental state". A practical method for grading the cognitive state of patients for the clinician. *Journal of psychiatric research.* Nov 1975;12(3):189- 198.**
5. **Green J, Thorogood N. *Qualitative methods for health research*: Sage; 2013.**
6. **Bazeley P, Jackson K, eds. *Qualitative data analysis with NVivo*: Sage Publications Limited; 2013.**
7. **Pettee Gabriel K, McClain JJ, Schmid KK, Storti KL, Ainsworth BE. Reliability and convergent validity of the past-week Modifiable Activity Questionnaire. *Public health nutrition.* Mar 2011;14(3):435-442.**
8. **Morisky DE, Green LW, Levine DM. Concurrent and predictive validity of a self-reported measure of medication adherence. *Medical care.* Jan 1986;24(1):67-74.**
9. **Hays RD, Kravitz RL, Mazel RM, et al. The impact of patient adherence on health outcomes for patients with chronic disease in the Medical Outcomes Study. *Journal of behavioral medicine.* Aug 1994;17(4):347-360.**
10. **Lejuez CW, Hopko DR, Hopko SD. A brief behavioral activation treatment for depression. Treatment manual. *Behavior modification.* Apr 2001;25(2):255-286.**
11. **Park LG, Howie-Esquivel J, Chung ML, Dracup K. A text messaging intervention to promote medication adherence for patients with coronary heart disease: a randomized controlled trial. *Patient Educ Couns.* Feb 2014;94(2):261-268.**
12. **Smith SC, Jr., Benjamin EJ, Bonow RO, et al. AHA/ACCF Secondary Prevention and Risk Reduction Therapy for Patients with Coronary and other Atherosclerotic Vascular Disease: 2011 update: a guideline from the American Heart Association and American College of Cardiology Foundation. *Circulation.* Nov 29 2011;124(22):2458-2473.**
13. **Eckel RH, Jakicic JM, Ard JD, et al. 2013 AHA/ACC guideline on lifestyle management to reduce cardiovascular risk: a report of the American College of Cardiology/American Heart Association Task Force on Practice Guidelines. *Circulation.* Jun 24 2014;129(25 Suppl 2):S76-99.**
14. **O'Gara PT, Kushner FG, Ascheim DD, et al. 2013 ACCF/AHA guideline for the management of ST-elevation myocardial infarction: a report of the American College of Cardiology Foundation/American Heart Association Task Force on Practice Guidelines. *Circulation.* Jan 29 2013;127(4):e362-425.**
15. **Thorndike AN, Regan S, McKool K, et al. Depressive symptoms and smoking cessation after hospitalization for cardiovascular disease. *Arch Intern Med.* Jan 28 2008;168(2):186-191.**
16. **Ziegelstein RC, Fauerbach JA, Stevens SS, Romanelli J, Richter DP, Bush DE. PAtients with depression are less likely to follow recommendations to reduce cardiac risk during recovery from a myocardial infarction. *Archives of Internal Medicine.* 2000;160(12):1818-1823.**
17. **Hayden-Wade HA, Coleman KJ, Sallis JF, Armstrong C. Validation of the telephone and in- person interview versions of the 7-day PAR. *Medicine and science in sports and exercise.* May 2003;35(5):801-809.**
18. **Mason B, Ross L, Gill E, Healy H, Juffs P, Kark A. Development and validation of a dietary screening tool for high sodium consumption in Australian renal patients. *J Ren Nutr.* Mar 2014;24(2):123-134 e121-123.**
19. **Thompson FE, Midthune D, Subar AF, Kipnis V, Kahle LL, Schatzkin A. Development and evaluation of a short instrument to estimate usual dietary intake of percentage energy from fat. *J Am Diet Assoc.* May 2007;107(5):760-767.**
20. **Cropsey KL, Trent LR, Clark CB, Stevens EN, Lahti AC, Hendricks PS. How low should you go? Determining the optimal cutoff for exhaled carbon monoxide to confirm smoking abstinence when using cotinine as reference. *Nicotine & tobacco research : official journal of the Society for Research on Nicotine and Tobacco.* Oct 2014;16(10):1348-1355.**
21. **Javors MA, Hatch JP, Lamb RJ. Cut-off levels for breath carbon monoxide as a marker for cigarette smoking. *Addiction.* Feb 2005;100(2):159-167.**
22. **Watson D, Clark LA, Tellegen A. Development and validation of brief measures of positive and negative affect: the PANAS scales. *Journal of personality and social psychology.* Jun 1988;54(6):1063-1070.**
23. **Spertus JA, Winder JA, Dewhurst TA, et al. Development and evaluation of the Seattle Angina Questionnaire: a new functional status measure for coronary artery disease. *Journal of the American College of Cardiology.* Feb 1995;25(2):333-341.**
24. **SF-12v2™: How to Score Version 2 of the SF-12® Health Survey. Lincoln, RI: QualityMetric Incorporated; 2002.**
25. **Hlatky MA, Boineau RE, Higginbotham MB, et al. A brief self-administered questionnaire to determine functional capacity (the Duke Activity Status Index). *The American journal of cardiology.* Sep 15 1989;64(10):651-654.**
26. **Menezes AR, Lavie CJ, Milani RV, Forman DE, King M, Williams MA. Cardiac rehabilitation in the United States. *Prog Cardiovasc Dis.* Mar-Apr 2014;56(5):522-529.**
27. **Hanssen TA, Nordrehaug JE, Eide GE, Bjelland I, Rokne B. Anxiety and depression after acute myocardial infarction: an 18-month follow-up study with repeated measures and comparison with a reference population. *European journal of cardiovascular prevention and rehabilitation : official journal of the European Society of Cardiology, Working Groups on Epidemiology & Prevention and Cardiac Rehabilitation and Exercise Physiology.* Dec 2009;16(6):651-659.**
28. **Spring B, King AC, Pagoto SL, Van Horn L, Fisher JD. Fostering multiple healthy lifestyle behaviors for primary prevention of cancer. *Am Psychol.* Feb-Mar 2015;70(2):75-90.**
29. **Joseph AM, Norman SM, Ferry LH, et al. The safety of transdermal nicotine as an aid to smoking cessation in patients with cardiac disease. *The New England journal of medicine.* Dec 12 1996;335(24):1792-1798.**
30. **Meine TJ, Patel MR, Washam JB, Pappas PA, Jollis JG. Safety and effectiveness of transdermal nicotine patch in smokers admitted with acute coronary syndromes. *The American journal of cardiology.* Apr 15 2005;95(8):976-978.**
31. **Woolf KJ, Zabad MN, Post JM, McNitt S, Williams GC, Bisognano JD. Effect of nicotine replacement therapy on cardiovascular outcomes after acute coronary syndromes. *The American journal of cardiology.* Oct 1 2012;110(7):968-970.**
32. **Paciullo CA, Short MR, Steinke DT, Jennings HR. Impact of nicotine replacement therapy on postoperative mortality following coronary artery bypass graft surgery. *Ann Pharmacother.* Jul 2009;43(7):1197-1202.**
33. **Cohen S, Kamarck T, Mermelstein R. A global measure of perceived stress. *J Health Soc Behav.***

**Dec 1983;24(4):385-396.**

1. **Kanter JW, Mulick, P. S., Busch, A. M., Berlin, K.S., & Martell, C. R. The Behavioral Activation for Depression scale (BADS): Psychometric properties and factor structure. *Journal of Psychopathology and Behavioral Assessment.* 2007;29:191-202.**
2. **Kanter JW, Rusch, L. C., Busch, A. M., & Sedivy, S. K. . Confirmatory factor analysis of the Behavioral Activation for Depression Scale (BADS) in a depressed sample. *Journal of*** ***Psychopathology and Behavioral Assessment.* 2009;31:36-42.**
3. **Busch AM, Leavens EL, Wagener TL, Buckley ML, Tooley EM. Prevalence, Reasons for Use, and Risk Perception of Electronic Cigarettes Among Post-Acute Coronary Syndrome Smokers. *J Cardiopulm Rehabil Prev.* Apr 26 2016.**
4. **Busch AM, Ciccolo JT, Puspitasari AJ, Nosrat S, Whitworth JW, Stults-Kolehmainen MA. Preferences for exercise as a treatment for depression. *Mental Health and Physical Activity.* In Press.**
5. Ziegelstein, R. C., Fauerbach, J. A., Stevens, S. S., Romanelli, J., Richter, D. P., & Bush, D. E. (2000). Patients with depression are less likely to follow recommendations to reduce cardiac risk during recovery from a myocardial infarction. *Archives of Internal Medicine*, *160*(12), 1818–1823.
